# Novel Antibacterial Agents SAAP-148 and Halicin Combat Gram-Negative Bacteria Colonizing Catheters

**DOI:** 10.3390/antibiotics12121743

**Published:** 2023-12-16

**Authors:** Nesrine Bouhrour, Tanny J. K. van der Reijden, Michella M. Voet, Bep Schonkeren-Ravensbergen, Robert A. Cordfunke, Jan Wouter Drijfhout, Farida Bendali, Peter H. Nibbering

**Affiliations:** 1 Laboratoire de Microbiologie Appliquée, Faculté des Sciences de la Nature et de la Vie, Université de Bejaia, Bejaia 06000, Algeria; 2 Department of Infectious Diseases, Leiden University Medical Center, 2300 RC Leiden, The Netherlands; t.j.k.van_der_reijden@lumc.nl (T.J.K.v.d.R.); m.m.voet@lumc.nl (M.M.V.); e.schonkeren@lumc.nl (B.S.-R.); nibbering@hhvbiotech.com (P.H.N.); 3 Department of Immunology, Leiden University Medical Center, 2300 RC Leiden, The Netherlands; r.a.cordfunke@lumc.nl (R.A.C.); j.w.drijfhout@lumc.nl (J.W.D.)

**Keywords:** catheter, antibiotic resistance, biofilm, persisters, halicin, SAAP-148

## Abstract

The antibiotic management of catheter-related infections (CRIs) often fails owing to the emergence of antimicrobial-resistant strains and/or biofilm/persister apparitions. Thus, we investigated the efficacy of two novel antimicrobial agents, i.e., the synthetic peptide SAAP-148 and the novel antibiotic halicin, against Gram-negative bacteria (GNB) colonizing catheters. The antibacterial, anti-biofilm, and anti-persister activities of both agents were evaluated against *Acinetobacter baumannii*, *Escherichia coli*, and *Klebsiella pneumoniae* strains. The enrolled strains were isolated from catheters and selected based on their resistance to at least three antibiotic classes and biofilm formation potential. Furthermore, the hemolysis and endotoxin neutralization abilities of these agents were explored. The bactericidal activity of both agents was reduced in urine and plasma as compared to buffered saline. In a dose-dependent manner, SAAP-148 and halicin reduced bacterial counts in 24 h preformed biofilms on silicone elastomer discs and eliminated persisters originating from antibiotic-exposed mature 7-day biofilms, with halicin being less effective than SAAP-148. Importantly, SAAP-148 and halicin acted synergistically on *E. coli* and *K. pneumoniae* biofilms but not on *A. baumannii* biofilms. The peptide, but not halicin, decreased the production of IL-12p40 upon exposure to UV-killed bacteria. This preliminary study showed that SAAP-148 and halicin alone/in combination are promising candidates to fight GNB colonizing catheters.

## 1. Introduction

Catheters are essential in the management of a range of clinical scenarios, such as the delivery of chemotherapy, antibiotics, and parenteral nutrition, as well as (hemo)dialysis [1,2]. Unfortunately, their use is complicated by their propensity to become colonized by bacteria, which may lead to serious infections [3,4], such as bloodstream and recurrent urinary tract infections, that affect significantly the length of hospital stays, mortality rates, and costs [5]. Catheter-related bloodstream infections (CRIs) are some of the most frequent, lethal, and costly complications of central venous catheterization [6], with incidences ranging from 1.8 to 5.2 per 1000 catheters [7]. In addition, urinary catheterization accounts for 40% of all the hospital-acquired infections in the United States [8] and possibly worldwide [9]. For many years, Gram-positive staphylococci were reported as the most common causative agents of CRIs, followed by Gram-negative bacteria (GNBs) [4,10,11,12]. However, in recent years, the epidemiology and microbiology of CRIs have changed, and a shift in the predominance of CRI pathogens from Gram-positive to GNBs, such as *Escherichia coli*, *Klebsiella pneumoniae*, and *Acinetobacter* spp., is widely reported [13,14,15,16,17]. These bacteria may originate from the endogenous flora of the patients (e.g., from mucosa) or from exogenous sources, such as other patients, healthcare workers, (hospital) environmental surfaces, or contaminated objects [18]. The treatment of patients with an infected catheter often involves the removal of the infected device, followed by intensive antibiotic therapy [19]. The failure of this treatment is caused by the emergence of antimicrobial-resistant strains [20,21] and/or the formation of biofilms [11,22] and persisters [23]. Upon the elimination of the stress, persisters revive and can start a new infection, which explains the persistence of these CRIs. Moreover, biofilms are considered as major contributors to persistent infections, constituting a global health problem [24]. Because of these considerations, novel antibacterial and anti-biofilm/persister agents for the treatment of CRIs are urgently required. Antimicrobial peptides (AMPs), which are molecules of the first line of defense against infections [25], are considered as promising candidates as they combine broad-spectrum antimicrobial activities with immune-modulating capabilities [26,27,28,29,30]. Interestingly, these peptides kill bacteria by mechanisms different from those of current antibiotics, such as interacting with and subsequently disrupting the microbial plasma membrane. Moreover, some AMPs can interact with the extracellular polysaccharides of the matrix, leading to the disintegration of biofilms [31,32]. One of the best-studied AMPs is human cathelicidin LL-37 [32,33,34,35], from which the LL-37-based synthetic antimicrobial and anti-biofilm peptide (SAAP)-148 was developed using random amino acid substitutions in the C-terminal part of LL-37 [28]. In a study by de Breij et al. [28], the peptide SAAP-148 was highly efficient in killing a panel of planktonic multidrug-resistant (MDR) bacteria, including colistin-resistant *E. coli*, *K. pneumoniae*, and *A. baumannii*. Further studies have also shown the efficacy of this peptide against biofilms [28,36] and persister cells [28,37,38]. de Breij et al. [28] investigated the mode of action of SAAP-148 and showed that the formation of pores in cell walls led to the permeabilization of the membrane, followed by the destruction of the bacterial cells. Recently, using computer-assisted deep learning discovery approaches (at the Drug Repurposing Hub) [39], halicin was identified as the first-in-class novel small molecule based on the probability of being able to inhibit *E. coli* growth. Importantly, additional assays demonstrated that halicin exhibits broad-spectrum antibacterial activities against clinical strains, such as carbapenem-resistant *Enterobacteriaceae*, MDR *A. baumannii* and *Pseudomonas aeruginosa*, *Mycobacterium tuberculosis*, and *Clostridium difficile*, in vitro and in a murine infection model [39]. Likewise, this agent was demonstrated as being effective against MDR *A. baumannii* 3086 [40] and 24 h immature and 7-day-mature biofilms formed on a polypropylene plate [36]. Moreover, it was suggested that the compound kills bacteria by dissipating the transmembrane electrochemical gradient, ∆pH, after binding with iron [39,41]. The effect of either SAAP148 or halicin on Gram-positive bacteria, especially *S. aureus*, has been extensively studied [28,36,37,38,40,42,43,44,45,46,47,48,49,50]. However, few reports on their effects on GNBs are available [28,36,39,40,46,47,48,49,51], and none of the studies tested strains isolated directly from medical devices and using both agents against GNBs. In this preliminary study, we investigated the antibacterial efficiencies of SAAP-148 and halicin as potential candidates for the development of a novel treatment for CRIs associated with GNBs. This study is the first one exploring in the same work the anti-biofilm and anti-persister activities of SAAP-148 and halicin alone and in combination against GNB strains isolated from catheters.

## 2. Results

### 2.1. Identification of the Bacterial Strains Colonizing Catheters

A total of 40 GNB strains isolated from 10 intravenous catheters (IVCs) and 4 urinary catheters (URCs) were identified using 16S rDNA sequencing. *Enterobacteriaceae*, predominantly *E. coli*, were the most abundant (81%) in the IVCs; the remainder (19%) was *Acinetobacter* spp., predominantly *A. baumannii* (Table 1). Similarly, all the bacterial strains isolated from the URCs were *Enterobacteriaceae*, e.g., *K. pneumoniae* (56%) and *E. coli* (44%) (Table 1).

### 2.2. Bacterial Resistance to Antibiotics

Virtually all the *E. coli* strains were sensitive to almost all the tested antibiotics, except for nalidixic acid (76%), tetracycline (34%), amoxicillin/clavulanic acid (24%), and cotrimoxazole (10%), while the *K. pneumoniae* isolates were resistant to β-lactams (except for cefoxitin), and two out of the five strains were resistant to gentamicin. The *Acinetobacter* isolates exhibited high levels of resistance to all the antibiotics, except for gentamicin (Table 1).

### 2.3. Bacterial Biofilm Formation and Virulence Gene Profiles

To further characterize these catheter-colonizing strains, their ability to form biofilms and the presence of genes involved in adherence to surfaces and biofilm formation were assessed. The results revealed that biofilm formation on polystyrene microplates varied widely among the *E. coli* (only 11/29 were strong formers) and *Acinetobacter* (only 2/6 were strong formers) strains, while all the *K. pneumoniae* strains formed significant biofilms (Table 1); 4/5 were strong producers. In addition, all 29 *E. coli* strains harbored the *fimH* gene; 28/29 strains, the *csgA* gene; and 15 out of the 29 strains (≈52%), the *hlyF* gene. All six *K. pneumoniae* strains harbored the *mrkD*, *fimH-1*, *ycfM*, and *ecpA* genes. In addition, all five *A. baumannii* strains exhibited the *ompA* and *csuE* genes, whereas four out of the five strains exhibited the *bap* gene (Table 1).

### 2.4. Bactericidal Efficacies of SAAP-148 and Halicin on Selected Strains

Based on the above characterization data, four GNBs (*E. coli* EC2, *A. baumannii* AB1, and *K. pneumoniae* KP1 and KP2), resistant to at least three antibiotics, were selected for further studies on the antibacterial properties of SAAP-148 and halicin. The results revealed that in a dose-dependent manner, SAAP-148 in PBS killed all four bacterial strains, with LC 99.9% values at concentrations ranging from sub-micromolar to low micromolar (Table 2). In addition, in a dose-dependent manner, the peptide was effective against these bacteria in 50% (*v*/*v*) pooled human urine and 50% (*v*/*v*) human plasma, although 4–8× higher peptide concentrations were required. Similar bactericidal activities were obtained after 4 and 24 h of exposure to the peptide. Also, in a dose-dependent manner, halicin killed all four bacterial strains in PBS, with LC 99.9% values ranging between 6.4 µM and 51.2 µM depending on the strain (Table 2). Halicin was more effective after 24 h than after 4 h of exposure. Of note, halicin was less effective against KP1, KP2, and EC2 in pooled urine than in PBS and virtually ineffective against AB1 in pooled plasma (Table 2). These results revealed that the peptide SAAP-148 and halicin are potent candidates as antibacterial drugs against GNBs.

### 2.5. Reduction in Bacterial Counts in Biofilms by SAAP-148 and Halicin

**Figure 1:**
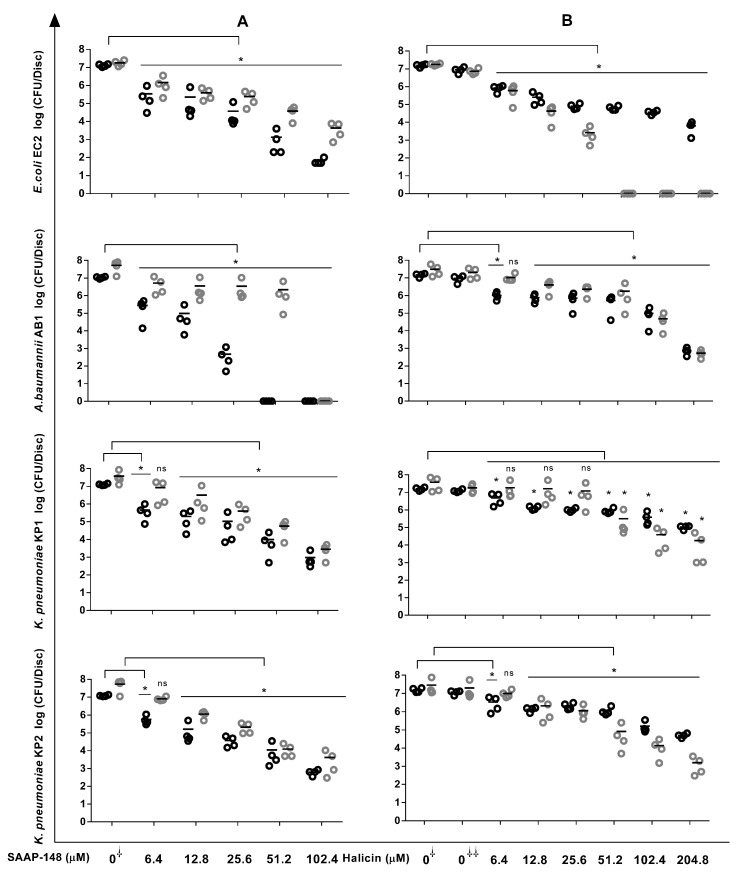
Reduction in bacterial counts within biofilms upon exposure to SAAP148 and halicin. In short, 24 h Gram-negative biofilms on silicone elastomer discs were exposed for 4 h and 24 h to increasing concentrations of SAAP-148 (**A**) and halicin (**B**); then, the biofilms were sonicated, and the number of surviving bacteria was assessed microbiologically. The results are expressed as the mean of the number of viable bacteria in log_10_ CFU/disc. Four experiments were undertaken in triplicate. The data obtained after 4 h and 24 h exposures are represented by open black and open grey symbols, respectively. Mann–Whitney U test: * *p* < 0.05 indicates significantly different from control (0^⸸^, no agent). DMSO control (0^⸸⸸^): control for halicin, i.e., the concentration of DMSO in the highest concentration of halicin that was tested. ns: not significantly different. Kruskal–Wallis test indicated that SAAP-148 as well as halicin at 4 h and 24 h were significantly effective against all the strains.

Because biofilms are central to CRIs, we next determined the abilities of SAAP-148 and halicin to eradicate 24 h biofilms on a silicone elastomer, a surface mimicking a catheter. After 4 h of exposure, the BBC99.9 values for the peptide against EC2, AB1, KP1, and KP2 significantly amounted to 51.2 µM, 25.6 µM, 102.4 µM, and 102.4 µM, respectively; while after 24 h of exposure, they were higher (>102.4 µM) (Figure 1). Identically, halicin was effective against the four-strain biofilm (BBC99.9 ≈ 102.4 µM). The agent was highly effective at a low concentration (~25.6 µM) against the EC2 biofilm compared with the other strains, which required high concentrations (~204.8 µM). These data indicated that the compounds could be promising anti-biofilm agents. 

### 2.6. Effects of SAAP-148 and Halicin on Persisters Derived from Antibiotic-Exposed Mature Biofilms

**Figure 2:**
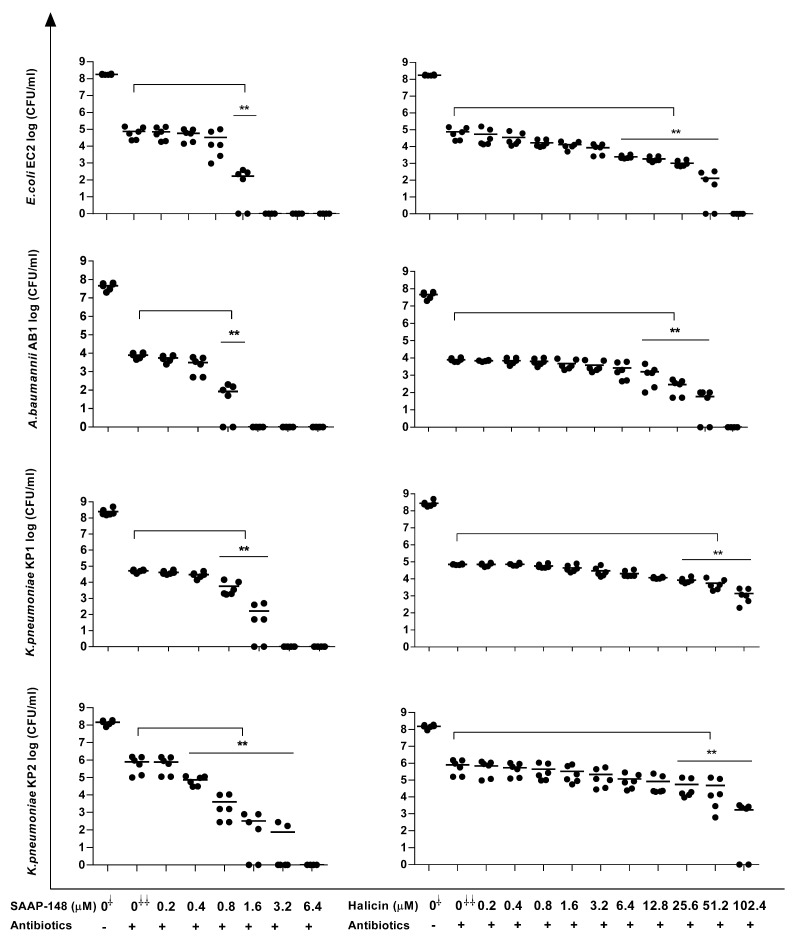
Effects of SAAP-148 and halicin on persisters derived from antibiotic-exposed mature biofilms. In short, bacterial biofilms were cultured for seven days, washed, exposed for 3 days to antibiotics, washed, and then exposed for 4 h to increasing concentrations of the peptide or halicin. Thereafter, the biofilms were sonicated to obtain a suspension of persisters, enabling the microbiological detection of the bacterial counts. The results are expressed as the mean of the number of viable bacteria in log_10_ CFU/mL. Three independent experiments in duplicate were undertaken. To enrich for persisters, *E. coli* EC2 and *K. pneumoniae* KP1 and KP2 were exposed to 50 × MBC ciprofloxacin; *A. baumannii* AB1, 50 × MBC gentamicin. The Kruskal–Wallis test indicated that SAAP-148 and halicin were significantly effective against all the persisters at 4 h. 0^⸸^: number of bacteria before treatment with antibiotic. 0^⸸⸸^: number of bacteria after 3 days of exposure to the antibiotic. ** *p* < 0.01 (Mann–Whitney U test) indicated significant differences from controls exposed to the antibiotic (0^⸸⸸^) but not SAAP-148/halicin.

Because persisters play a pivotal role in chronic infections and antibiotic treatment failure, we assessed the effects of SAAP-148 and halicin on persisters derived from 7-day mature biofilms that had been exposed to high doses of antibiotics for an additional three days. As expected, high doses (50 × MBC) of ciprofloxacin significantly (*p* < 0.01) reduced (≥ 100×) the numbers of viable bacteria in the EC2, KP1, and KP2 biofilms, as did similarly high doses of gentamycin for the bacterial counts in the AB1 (*p* < 0.01) biofilms, without the complete elimination of all the bacteria (Figure 2). However, SAAP-148 was highly effective in killing persisters derived from antibiotic-exposed mature EC2, AB1, KP1, and KP2 biofilms (Figure 2), with the complete eradication of the EC2 and AB1 persisters at 1.6 µM. For KP1 and KP2, slightly higher concentrations, i.e., 3.2 µM and 6.4 µM of the peptide were required, respectively (Figure 2). Interestingly, the EC2 and AB1 persisters were eliminated by high concentrations of halicin, whereas the KP1 and KP2 persisters were not eradicated by this agent (Figure 2).

### 2.7. SAAP-148 and Halicin Neutralize GNB-Induced IL-12p40 Production by Human Blood Leukocytes

**Figure 3:**
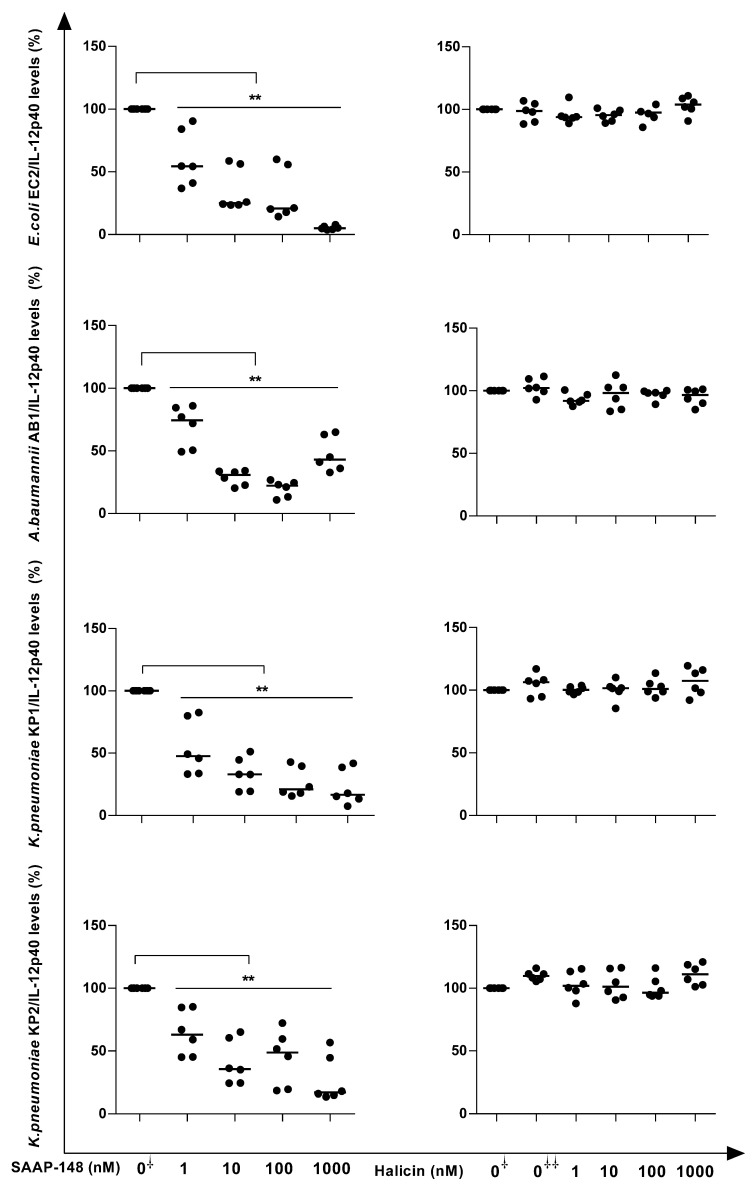
Pre-incubation for 2 h of UV-inactivated GNB strains with SAAP-148, but not halicin, reduced the ability of GNBs to induce IL-12-p40 production by whole human blood leukocytes. In short, UV-killed bacteria were pre-incubated with increasing concentrations of SAAP-148 or halicin (from 1 to 1000 nM) for 2 h and then mixed with 5-fold diluted whole blood. After 20 h of incubation at 37 °C, the cells were spun down, and the levels of IL-12p40 in the supernatants were assessed using ELISA. The results are expressed as the percentage of IL-12p40 compared with that of the control (0^⸸^), i.e., UV-inactivated bacteria exposed to PBS instead of the peptide. 0^⸸⸸^: UV-inactivated bacteria exposed to the highest concentration of DMSO instead of the halicin concentration that was tested. Values are the means of three independent experiments, each performed in duplicate. The Kruskal–Wallis test indicated that SAAP-148 significantly reduced the abilities of all the bacteria (EC2 and AB1: *p* < 0.0001; KP1: *p* = 0.0004 and KP2: *p* = 0.0005) to induce IL-12p40 production. ** *p* < 0.01 compared to the control (no peptide) using Mann–Whitney U test.

We compared the abilities of SAAP-148 and halicin to reduce the capacities of EC2, AB1, KP1, and KP2 to stimulate the production of the pro-inflammatory cytokine IL-12p40 by human blood leukocytes. The results revealed that in a concentration-dependent manner, the pre-incubation of UV-inactivated bacteria with SAAP-148, but not halicin, reduced the ability of the strains to stimulate IL-12p40 production by blood leukocytes (Figure 3).The concentrations of SAAP-148 that significantly (*p* < 0.05) reduced the abilities of UV-inactivated EC2, AB1, KP1, and KP2 to induce cytokine production amounted to ≥1 nM, ≥10 nM, ≥10 nM, and ≥10 nM, respectively (Figure 3). These preliminary data revealed that the synthetic peptide exhibited promising activity as an anti-inflammatory agent. 

### 2.8. Hemolytic Activities of SAAP-148 and Halicin

**Figure 4:**
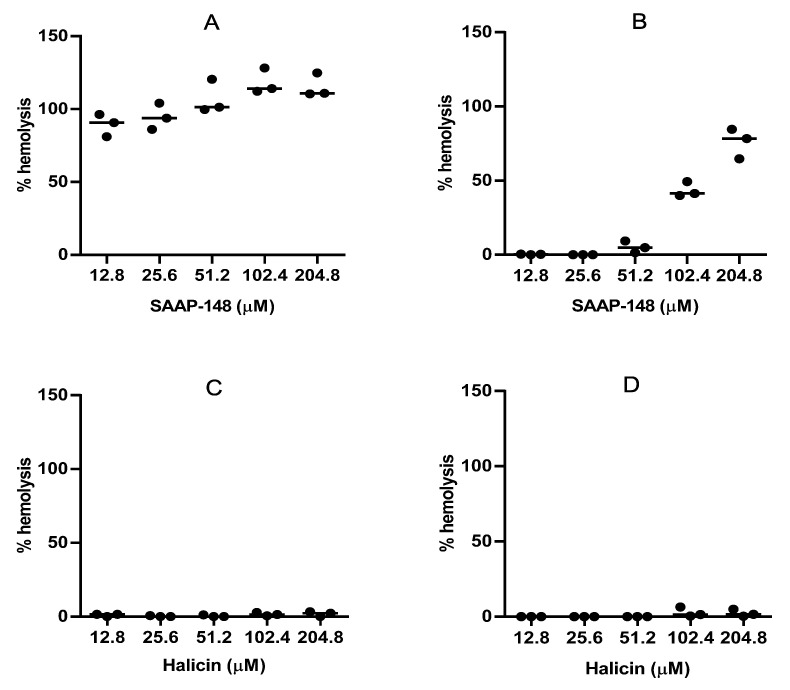
Hemolytic activities of SAAP-148 in PBS (**A**) and 50% plasma (**B**) and of halicin in PBS (**C**) and 50% plasma (**D**). Values are means of three independent experiments, each performed in triplicate.

To obtain some insight into the in vitro toxicities of SAAP-148 and halicin, we assessed the hemolytic activities of these compounds using human erythrocytes suspended in 50% (***v*/*v***) human plasma and in PBS. The highest concentrations of SAAP-148 that lysed ≤5% of the erythrocytes in the PBS and 50% plasma amounted to <12.8 µM and 51.2 µM (Figure 4A,B), respectively. The hemolytic activity of the halicin amounted to ≥204.8 µM in the PBS and 50% plasma (Figure 4C,D, respectively), indicating that halicin is less hemolytic than the peptide.

### 2.9. Interactions between SAAP-148 and Halicin

As the modes of action underlying the antibacterial activities of SAAP-148 and halicin differ [28,39], the possibility of synergistic and/or additive interactions between these novel agents in eradicating bacteria in biofilms was investigated using checkerboard assays. The results revealed that several combinations of SAAP-148 and halicin were more effective than each agent alone in reducing bacterial counts in EC2, KP1, and KP2 biofilms but not in AB1 biofilms (Table 3). Based on the fractional biofilm eradication concentration index (ΣFBEC), some combinations of SAAP-148 and halicin acted synergistically on bacteria in EC2, KP1, and KP2 but not AB1 biofilms, whereas other combinations exerted additive effects (Table 3). Importantly, the concentrations of SAAP-148 needed to eliminate the bacteria in biofilms in the presence of halicin were considerably lower than that required for the peptide alone.

## 3. Discussion

The present study is the first undertaken in Bejaia (in the northeast of Algeria) and, thus, constitutes a precious source of information for the region and the country. As recently reported, a predominance of GNBs (40 out of 50 catheter-colonizing strains) was observed rather than Gram-positive ones, which explains our focus on these bacteria in this study. Furthermore, according to Buetti et al. [53], most epidemiological studies so far have neglected to focus on GNBs as causes of catheter infections. *E. coli* (~81%) and *A. baumannii* (16%) were the predominant species in the intravenous catheters, and this could be explained by the local prevalence of these ubiquitous pathogens in the hospital environment [54,55,56,57,58]. It is well documented that the hospital environment (e.g., bed rails, mattresses, medical equipment, colonized or infected patients, and the hands of healthcare workers) is an ecological niche for *A. baumannii* and *E. coli* [59,60,61]. For urinary catheters, *K. pneumoniae* was the most isolated species in this study (~56%), followed by *E. coli* (44%), which is consistent with the results found by Barbadoro et al. [62] but not by others [21,63,64,65]. In Algeria, the predominance of *E. coli* in urinary tract infections is widely reported [66,67,68], but no epidemiological data are available for catheter-colonizing uropathogens. It is not surprising to find these two uropathogens because *E. coli* and *K. pneumoniae* are fecal contaminants or residents of patients’ native or transient microflora that colonize the peri-urethral area [69].

Another observation pertains to the relation between the biofilm formation and presence of genes related to this process. As all the bacteria were derived from colonized catheters, we expected 100% of the strains to form a biofilm on polystyrene surfaces. However, if this was true for *K. pneumoniae* isolates (100%), only 83% (24/29) of the *E. coli* and 83.3% (5/6) of the *Acinetobacter* isolates were biofilm formers, which is in agreement with other reports [70,71,72]. Of course, it cannot be excluded that the characteristics of the surface play a role in biofilm formation. Therefore, in further experiments, we used silicone elastomers, which more accurately mimic medical devices, such as venous and urinary catheters [73]. Biofilm formation is mediated by multiple virulence factors, such as fimbriae (*fimH*) and curli (*csgA*), for *E. coli* [74]; CsuE, a subunit of the chaperone–usher pili (Csu), the outer membrane protein (OmpA), and the biofilm-associated protein (Bap), for *A. baumannii* [75,76,77]; and fimbriae type 1 (*fimH-1*) and type 3 (*mrkD*), for *K. pneumoniae* [78]. In the present study, these genes were present in all the strains. However, a comparison between the biofilm-forming and non-biofilm-forming isolates revealed that the presence of the virulence factors was not associated with biofilm production. 

Surprisingly, the *E. coli* strains were sensitive to almost all the antibiotics that were tested. These results are not in agreement with other Algerian studies reporting a prevalence of high resistance to β-lactams, gentamicin, and ciprofloxacin, except for imipenem, which was active against *E. coli* strains [67,79,80,81]. Our results showed that a high rate of biofilm-forming *E. coli* strains (83%) was more sensitive to antibiotics, indicating no clear correlation between the ability to form a biofilm and sensitivity to antibiotics. This can be explained by the fact that the strains form biofilms to survive when exposed to antibiotics [82]. Another explanation could be that the polymeric matrix acts as a barrier and protects the bacteria from antibiotics, thereby preventing the penetration of the biofilm by the antibiotics. In addition, the binding of antibiotics to matrix components may reduce the activities of the antibiotics [83,84,85]. Alves et al. [86] reported that 73% of positive biofilm-forming *E. coli* isolates were sensitive to antibiotics. Poursina et al. [87] also concluded that non-MDR *E. coli* strains were able to form strong biofilms. The sensitivity of the strains to imipenem could be explained by its rare use in Algerian hospitals. We found that the *K. pneumoniae* strains harbored high rates of resistance to amoxicillin/clavulanic acid and ß-lactams (expect for cefoxitin), tetracycline, and gentamycin, while all the strains were sensitive to imipenem, nalidixic acid, ciprofloxacin (except for the KP5 strain), and cotrimoxazole. The majority of the Algerian studies used GNB clinical isolates, but a few Algerian studies enrolled catheter-colonizing strains. High resistance to antibiotics has also been reported in other countries [78,88,89,90,91]. The *Acinetobacter* spp. strains exhibited high resistances to ß-lactams (expect for cefoxitin), imipenem, tetracycline, ciprofloxacin, and cotrimoxazole, but no strain was resistant to gentamycin, which is in agreement with antibiotic resistances in *A. baumannii* strains reported by other Algerian researchers [55,80,92,93]. 

It must be noted that the main objective of this study was the evaluation of the antibacterial and anti-biofilm effects of SAAP-148 and halicin alone or in combination on GNB-colonizing catheters. We used single strains of *E. coli* and *A. baumannii* and two *K. pneumoniae* strains. The results of the killing assay revealed that SAAP-148 and halicin are highly active against the tested strains. This observation is in agreement with earlier reports showing the effectiveness of SAAP-148 [28] and halicin [39] against a wide range of bacteria, including MDR GNBs (*E. coli*, *A. baumannii*, *P. aeruginosa*, and *K. pneumoniae*). Contrariwise, the activities of both agents were reduced in human plasma and urine. As already reported, the antimicrobial activities of peptides and antibiotics could decrease in the presence of these physiological fluids owing to the presence of components preventing the interaction of these agents with bacterial membranes [94,95]. In addition, SAAP-148 and halicin significantly reduced the bacterial counts within biofilms, and the required concentrations were considerably higher than those required for directly killing planktonic cells. This can be attributed to the biofilm matrix, which acts as a barrier that delays or prevents the interaction of antimicrobial agents with bacterial cells, thereby reducing the effectiveness of the agents against the biofilms [96]. The results obtained with SAAP-148 are consistent with previous studies showing its efficacy against GNB biofilms [28,36]. Notably, compared with the study by van Gent et al. [36], where a high concentration (68.3 µM) of halicin was needed, this study revealed that halicin displayed the strongest effect on the *E. coli* biofilm at a low concentration (~25 µM). These findings suggest that the compounds have a promising anti-biofilm activity. 

The presence of persister cells, which can survive in up to high doses of antibiotics, is mainly responsible for chronic infections and their recalcitrance [97]. In the present work, we exposed mature biofilms to high concentrations (50 × MBC) of ciprofloxacin (EC2, KP1, and KP2) and gentamycin (AC1) for 3 days. These antibiotics induced a deep disruption of the matrix, leading to their penetration of the biofilms [98,99] and, thus, significantly reducing the number of bacteria within them. It has been suggested that the decrease in the activities of these antibiotics is correlated with persister cells [82]. SAAP-148 and halicin were effective against these persisters derived from the antibiotic-exposed mature biofilms of the four bacterial strains tested in a dose-dependent manner. This is in agreement with earlier findings that SAAP-148 effectively reduced bacterial counts within antibiotic-exposed mature biofilms of MRSA [37,38]. Herein, we provided proof for the first time that SAAP-148 possesses a potent anti-persister effect against GNB strains. Further, it should be kept in mind that planktonic, sessile, and persister bacteria all play important roles in CRIs, including catheter-related bloodstream infections and recurrent urinary tract infections [100,101]. 

The development of molecules able to kill GNBs without releasing their endotoxins (LPS), which are located in the outer membrane and induce severe pro-inflammatory responses, is a major challenge [102,103]. Peptides are able to bind to LPS and act as anti-inflammatory agents against endotoxic shock [104,105]. In our study, we found that SAAP-148, but not halicin, was able to reduce the ability of UV-killed GNBs to induce IL-12-p40 cytokine production in vitro. Furthermore, these results are reported for the first time, indicating that this synthetic peptide could be an excellent candidate as an anti-inflammatory agent (in vitro) to treat sepsis caused by GNB infections. In vivo experiments to monitor organ injury in mouse models of LPS-induced endotoxemia and *GNB*-induced septic shock could give more evidence about the efficiency of SAAP-148, as reported in research by Jang et al. [102]. Several hypotheses on the mechanism of action of peptides that can induce an anti-inflammatory response have been reported. First, cationic peptides bind to LPS via electrostatic interactions, thereby neutralizing anionic amphiphilic lipid A. Second, the peptide binds to the macrophage’s CD14 receptor and competitively inhibits the interaction of the LPS–LPB complex and, thus, blocks access to the TLR4 receptor, which mediates pro-inflammatory cytokines. Third, most LPS-binding peptides are able to dissociate LPS oligomers by depolymerization, thereby inhibiting LPS from binding to LBP [106,107]. 

In addition to antibacterial, anti-biofilm, and anti-inflammatory activities, low toxicity against human red blood cells (RBCs) is an important parameter for novel agents to be used as potential candidates in the treatment of biofilm-related infections [108]. We demonstrated that compared with halicin, which has no effect on RBCs, the peptide SAAP-148 is more toxic toward erythrocytes. The cytotoxicity assay, which is not included herein but is reported in a study by van Gent et al. [36], also showed that SAAP-148 was more cytotoxic than halicin for skin fibroblasts and RT-4 urethelial cells. It is known that hydrophobicity is crucial for peptides to disintegrate bacterial membranes. It has been reported that peptides possessing high hydrophobicity are correlated with high toxicity [36,109]. The increasing hydrophobic content (tryptophan, lysine, and arginine) leads to a significantly high hemolytic activity [110,111]. Moreover, SAAP-148 possesses a larger hydrophobic region in its amino-acid sequence [112]. The ability to form amphipathic structures has also been related to increased hemolytic activity [113]. Using SAAP-148 in combination with other agents could reduce the concentration of the peptide, thereby reducing the peptide’s toxicity. Interestingly, recent studies have reported improved strategies for designing potent and safe peptides for therapeutics, such as PLGA (poly(lactic-co-glycolic acid)) nanoparticles [49] and C-terminal PEGylation [47], which seem to be promising approaches to overcome these drawbacks. 

Furthermore, synergistic and additive interactions were noted between SAAP-148 and halicin against *E. coli* and *K. pneumoniae*, but not *A. baumannii*, in biofilms. These findings are in agreement with previous observations that the peptide and halicin act synergistically against planktonic MRSA but not against MDR *A. baumannii* [36]. Unfortunately, we cannot give an explanation for these species/strain-specific interactions between SAAP-148 and halicin. However, other researchers have reported similar findings for SAAP-148 and classical antibiotics [42] and for other antimicrobial peptides combined with other antimicrobial agents against GNB biofilms [114,115,116,117,118]. Importantly, synergistic combinations of the peptide and halicin enable the use of lower doses of SAAP-148, possessing good antibacterial activities, while limiting harmful side effects in vivo. The use of antimicrobial peptides in combination with conventional/novel antibiotics or other antimicrobial agents could be an effective strategy to reduce the development of resistance [119]. Future medical applications of (combinations of) SAAP-148 and halicin can be both invasive and non-invasive. Of note, combination treatment faces several hurdles, including the cytotoxicity and poor PK/PD properties of SAAP-148. However, conjugates of SAAP-148 and halicin could overcome several limitations encountered by the combinations. Regarding non-invasive usage, (combinations or conjugations of) SAAP-148 and halicin may be considered for antibiotic lock therapy [120,121], where (i) the catheter can be coated with the agents encapsulated in a polymeric formulation, such as hydrogel-loaded peptides and peptide-releasing hydrogels [122,123,124], or (ii) the agents may be immobilized on the surface of the catheter [122]. The use of antimicrobial agents as a coating is the most popular approach owing to their ability to target microorganisms in different ways [125]. Several studies have reported the effectiveness of the use of antibiotics [126,127,128] and antimicrobial peptides [129,130] as coatings for eradicating/preventing the colonization of pathogens on catheter surfaces. Alternatively, the agents may be used invasively, e.g., immediately after the removal of a colonized catheter or as a treatment for patients with a CRI in whom the current treatment has failed. 

It should be mentioned that the current in vitro study has some limitations. First, all the experiments were performed on a single strain of *E. coli* and *A. baumannii* and two *K. pneumoniae* strains from one hospital. Another limitation of this study is that the experiments were performed under static conditions (microtiter plate assays), whereas colonized catheters in situ are constantly subjected to a dynamic flow of blood or urine. This limitation may be circumvented in future experiments using flow cells, e.g., a modified Robbin’s device, in which a biofilm is formed in an environment of plasma or urine flowing under a constant shear (reviewed by Subramanian et al. [131]). 

## 4. Materials and Methods

### 4.1. Isolation of Bacteria from Catheters

Between February and April 2016, 14 catheters (4 urinary and 10 intravenous catheters) were removed from 14 patients (5 women and 9 men) hospitalized for at least 48 h in the departments of resuscitation, general surgery, and internal medicine at Bejaia University Hospital (Bejaia, Algeria) because of infectious disease complications. The age of the patients ranged from 27 to 96 years, and the duration that the catheter was *in situ* ranged from 2 to 16 days. The bacteria were harvested from the catheters using the method of Brun Buisson et al. [132] with minor modifications. For this purpose, the tip of the catheter was cut off, transferred to 10 mL of a sterile tryptone salt solution (TS; 0.1% *w*/*v*), and then sonicated for 5 min at 42 kHz (ultrasonic cleaner, Bransonic, Saint Louis, MO, USA). The bacterial suspension was vortexed for 1 min, after which the bacteria were spread on nutrient agar (NA, Conda, Madrid, Spain) and trypticase soy agar (TSA, Biokar Diagnostics, Allonne, France) plates for the enumeration of the total flora and on eosin methylene blue agar (EMB, Conda, Madrid, Spain) plates to assess the *Enterobacteriaceae* counts. The remaining catheters were washed and resuspended in 10 mL of nutrient broth (NB, Conda, Madrid, Spain). After incubation at 37 °C for 24–48 h, streak isolations were prepared from each positive broth on the specific agar plates mentioned above. The bacteria were identified using conventional methods. 

### 4.2. Identification of the Strains

All the Gram-negative isolates were genotypically identified using 16S rDNA sequencing. Briefly, single colonies were obtained from the NA plate using a sterile plastic disposable loop, and bacterial DNA was extracted using 20 µL of lysis buffer (0.25% (*w*/*v*) SDS, 0.05 N NaOH) and by heating for 15 min at 95 °C. The DNA samples were rapidly cooled and centrifuged at 13,000 rpm for a few seconds (Beun De Ronde, Abcoude, the Netherlands). Next, 180 µL of milli-Q water was added, and the DNA solution was mixed thoroughly, centrifuged again for 5 min at 13,000 rpm, and stored at −20 °C until use. PCR was used to amplify approximately 1500 bp of a consensus 16S rDNA gene: forward primer αβ NOT (5′-AGT TTG ATG CTG GCT CAG-3′) and reverse primer ω MB (5′-TAC CTT GTT ACG ACT TCG TCC CA -3′). Two microliters of the DNA sample, 2 µL of the 10 µM forward and reverse primers, 25 µL of the master mix (Promega, Madison, WI, USA), and 21 µL of milli-Q water were mixed and subjected to the following program: 1 × 5 min at 95 °C; 3 × 1 min at 95 °C; 2 min at 50 °C; 1 min at 72 °C; 35 × (45 s at 95 °C, 45 s at 50 °C, and 1 min at 72 °C); and 1 × 5 min at 72 °C. The amplified products were analyzed using electrophoresis (Bio-Rad, Hercules, CA, USA) on 1% (*w*/*v*) agarose (Roche Diagnostics, Mannheim, Germany) gel at 120 V for 2 h. The PCR product was cleaned as follows: 0.5 µL of *ExoI* (20 units/µL; Thermo Scientific, Vilnius, Lithuania) and 1 µL of *Fast AP* (1 unit/µL; Thermo Scientific) were added to 5 µL of the PCR product and incubated at 37 °C for 15 min. The enzymes were then deactivated by heating at 85 °C for 15 min, and the mixture was finally diluted 10× with Milli-Q water. For sequencing, 5 µL of the PCR product, 1 µL of the forward and reverse primers (1 pmol/µL) and 4 µL of Milli-Q water were mixed. The sequencing was performed on an Applied Biosystems 96-capillary system, ABI3730xl with a pop7 matrix (Macrogen, Amsterdam, the Netherlands).

### 4.3. Antibiotic Susceptibility Testing

The antibiotic susceptibility of the strains was tested using the disc diffusion method [133]. Briefly, bacterial suspensions of 0.5 McFarland were seeded on Mueller–Hinton agar (Becton Dickinson, Mississauga, ON, Canada) plates. The plates were air-dried for 15 min and then the discs impregnated with antibiotics were deposited on the plates (antibiotics in Supplementary Table S1). The diameter of the inhibition zone around each disc was measured after 24 h at 37 °C, and the strains were graded as sensitive (S), intermediate (I), or resistant (R) following the European Committee on Antimicrobial Susceptibility Testing guidelines [134]. *Escherichia coli* ATCC25922 was included as a reference strain for quality control.

### 4.4. *Detection* of Bacterial Virulence and Adhesion Genes 

The presence of virulence and adhesion genes in the clinical isolates was assessed using PCR. Briefly, 50 μL of a buffer containing bacterial DNA; 25 μL of the master mix (Taq polymerase, polymerase buffer (pH 8.5), and 400 µM dNTPs2x (Promega)); and 10 µM forward and reverse primers (specific primers in Supplementary Table S2) were mixed. The PCR programs are indicated in Supplementary Table S3.

### 4.5. Biofilm Formation on Polystyrene Microplates

The biofilm formation on sterile flat-bottom 96-well polystyrene microplates (Greiner Bio-One, Frickenhausen, Germany) was assessed using the method by O’Toole [135]. The wells of the microplate, previously filled with 100 µL of trypticase soy broth (TSB, Biokar Diagnostics), were seeded in triplicate with 100 µL of an 18 h bacterial culture (1 × 10^6^ CFU/mL) and incubated at 37 °C for 24 h. Wells containing 200 μL of sterile TSB broth were included as negative controls. After incubation, the cultures were removed; the wells were washed carefully with 200 μL of sterile TS solution, and the biofilms were fixed with 200 μL of absolute ethanol (Biochem Chemopharma, Montreal, Quebec, Canada). Subsequently, the fixed biomass was washed, stained with 0.1% (*w*/*v*) crystal violet solution (Biochem Chemopharma) for 30 min, and finally washed with 200 μL of TS solution. The biomass was finally quantified using a microplate reader (Chromate Awareness Technology, Palm City, FL, USA) to measure the absorbance at 630 nm for the crystal violet extracted with 96% (*v*/*v*) ethanol. The results were expressed as mean values.

### 4.6. Novel Antibacterial Agents

The SAAP-148 peptide (amide-LKRVWKRVFKLLKRYWRQLKKPVR-acetyl; Mw 3970) was synthesized using 9H-fluorenylmethyloxycarbonyl (Fmoc) chemistry in an automated peptide synthesizer (Syro II, MultiSyntech, Witten, Germany), as described previously [28,136]. The molecular mass of the peptide was confirmed using mass spectrometry (Voyager DE-Pro, PerSeptive Biosystems, Framingham, MA, USA), and the purity amounted to >95%, as determined using reverse-phase high-performance liquid chromatography (HPLC) and detection at 214 nm [137]. The lyophilized peptide was stored at −20 °C until use. A stock solution of 5.12 mM SAAP-148 was prepared in Milli-Q water and stored in the refrigerator at 4 °C. Halicin (SU 3327; Mw 261.3) was purchased from Tocris Bioscience (Bristol, UK). Prior to use in the experiments, the halicin was dissolved in 748 µL of dimethyl sulfoxide (DMSO, Honeywell Riedel-de Haën, Seelze, Germany) and further diluted in phosphate-buffered saline (PBS, 140 mM, pH 7.4; Fresenius KABI, Zeist, the Netherlands) to a stock solution of 51.2 mM.

### 4.7. In Vitro Killing Assay

From glycerol stock suspensions of *K. pneumoniae* (KP1 and KP2), *A. baumannii* (AB1), and *E. coli* (EC2), overnight cultures were prepared at 37 °C. Thereafter, the bacteria were cultured to the mid-log phase in TSB (Oxoid, Basingstoke, UK) for 2.5 h under continuous rotation, centrifuged at 3000 rpm for 10 min, and resuspended in PBS to a concentration of 5 × 10^6^ CFU/mL. Subsequently, 30 μL of PBS containing SAAP-148 or halicin (final concentrations ranging from 0.8 to 102.4 μM) and 2% (*v*/*v*) bacterial suspension were mixed with pure PBS; PBS supplemented with 1% (*v*/*v*) TSB; or PBS supplemented with 50% (*v*/*v*) pooled human plasma (for AB1) or 50% (*v*/*v*) pooled urine (for KP1, KP2, and EC2) in the wells of a polypropylene V-shape microplate (Greiner bio-one). After incubation for 4 h and 24 h at 37 °C (200 rpm), the number of viable bacteria was assessed microbiologically. The results were expressed as lethal concentration (LC) 99.9, i.e., the lowest concentration of the compound that killed 99.9% of the *inoculum*.

### 4.8. Anti-Biofilm Assay

One-hundred microliters of a suspension of log-phase bacteria (approximately 10^7^ CFU/mL in brain heart infusion (BHI) broth, Oxoid, Basingstoke, UK) were added to each well of a flat-bottom 96-well polystyrene microplate containing a sterile silicone elastomer disc (Ø = 4 mm), which mimics medical devices, like intravenous and urinary catheters, and incubated for 24 h at 37 °C. Thereafter, each disc was gently transferred in a sterile 96-well microplate, washed with PBS, and transferred again in a sterile flat-bottom 96-well polystyrene plate for exposure to increasing concentrations of SAAP-148 (in the range 6.4–102.4 µM) or halicin (in the range 6.4–204.8 µM) for 4 h and 24 h at 37 °C. Three discs were incubated with PBS as controls. In addition, three discs were exposed to 0.4% (*v*/*v*) DMSO. The concentration of DMSO in the highest concentration of halicin that was tested served as a control for halicin. After the treatment, each disc was washed as described above; transferred to a sterile, flat-bottom, 96-well polystyrene microplate containing 100 µL of PBS; and sonicated for 10 min at 42 kHz. The number of surviving bacteria was determined microbiologically. The results were expressed as biofilm bactericidal concentrations (BBC99.9), i.e., the lowest concentrations of the agents resulting in a 99.9% reduction in the bacterial counts in the biofilms.

### 4.9. Checkerboard Assay for Determination of SAAP-148 and Halicin Synergy

A checkerboard assay was used to determine the possible synergy between SAAP-148 and halicin against bacteria in biofilms. In short, 24 h biofilms formed on silicone elastomer discs, as described above, were washed two times with 100 µL of PBS and then exposed to combinations of increasing doses of SAAP-148 and halicin (Supplementary Table S4) for 24 h at 37 °C. Thereafter, the antimicrobial agents were gently removed; the plates were washed two times with PBS and sonicated for 10 min at 42 kHz. The number of surviving bacteria was determined microbiologically. The fractional biofilm eradication concentration index (ΣFBEC) was calculated according to the following formula: ΣFBEC = FBEC_A_ + FEBC_B_ [138], where FBEC_A_ = MBEC of compound A in the combination/MBEC of compound A, and FBEC_B_ = MBEC of compound B in the combination/MBEC of compound B. Synergy was defined as ΣFBEC ≤ 0.5; additive effect, 0.5 < ΣFBEC ≤ 1; indifference, 1 < ΣFBEC ≤ 2; antagonism, ΣFBEC > 2.

### 4.10. Anti-Persister Assay

Seven-day mature biofilms were developed in flat-bottom, 96-well polypropylene plates using 100 µL of 1 × 10^7^ CFU/mL bacteria in BHI (Oxoid) broth. The plates were sealed and incubated at 37 °C in a humidified atmosphere for 7 days. Thereafter, non-adherent bacteria were removed by two washes with PBS, and 100 µL of fresh BHI broth containing 50 × MBC of ciprofloxacin (25 µg/mL; Sigma-Aldrich, St. Louis, MO, USA) for KP1, KP2, and EC1 and 50 × MBC of gentamycin (400 µg/mL; Sigma-Aldrich) for AB1 were carefully added to each well. The medium containing the antibiotics was refreshed daily for 3 days. After the treatment, the supernatant was removed; the biofilm was sonicated at 42 kHz for 10 min, and the resulting bacterial suspensions from 48 wells were pooled. A total volume of 10 µL of increasing doses of SAAP-148 or halicin (in the range 0.2–102.4 µM) was added to 90 µL of the pooled bacterial suspension and incubated for 4 h at 37 °C (and 200 rpm). As a control, bacteria were exposed to PBS without SAAP-148/halicin. Afterward, the number of viable bacteria was determined microbiologically. To exclude the possibility that persisters surviving the treatment were missed, the incubation on the agar plates was prolonged for up to 5 days.

### 4.11. Assay for Bacterial Endotoxin Neutralization Capacities of SAAP-148 and Halicin 

Approximately 15 µL of UV-inactivated (for 20 min; ChemiDoc, Bio-Rad, Hercules, California, USA) bacteria (1 × 10^7^ CFU/mL in PBS) were mixed with 15 µL of SAAP-148 or halicin at final concentrations ranging from 1 to 1000 nM in a 96-well polypropylene V-bottom microplate and incubated for 2 h at 37 °C. Thereafter, 120 µL of Roswell Park Memorial Institute medium (RPMI, Thermo Fisher, Paisley, UK) was added to each well to dilute the samples 5×. Next, 5 µL of this mixture was added to 195 µL of 5× diluted heparinized blood obtained from healthy donors, and the mixture was incubated for 20 h at 37 °C under 5% CO_2_. Subsequently, the plates were spun for 5 min at 1200 rpm; the supernatants were aliquoted and stored at −20 °C until further analysis. The level of the pro-inflammatory cytokine IL-12p40 in these supernatants was quantified using the BioLegend Elisa max deluxe set (BioLegend, San Diego, CA, USA) according to manufacturer’s instructions, and the optical density (OD) at 450 nm was measured on a Spectramax i3X (Molecular Devices, Wokingham, UK).

### 4.12. Hemolysis Assay 

The hemolytic activities of the peptide SAAP-148 and halicin were investigated. Briefly, freshly drawn citrate blood, from healthy volunteers, was washed three times with PBS by centrifugation at 1000 rpm for 10 min and then resuspended in PBS to prepare an erythrocyte suspension of 20% (*v*/*v*). Subsequently, 25 μL of PBS containing SAAP-148 or halicin (at final concentrations ranging from 12.8 to 204.8 μM) and 2% (*v*/*v*) erythrocyte suspension were mixed with 50 μL of PBS or pooled human plasma in a 96-well polypropylene V-bottom plate (Greiner bio-one), shaken for 10 sec at 300 rpm, and incubated for 1 h at 37 °C. DMSO and 5% (*v*/*v*) Triton-X (Fluka Chemie, Buchs, Switzerland) were used as controls for 0% and 100% hemolysis, respectively. After centrifugation for 3 min at 1200 rpm, the supernatant was carefully transferred to new 96-well, polypropylene flat-bottom plates, and the hemoglobin release was determined by measuring the OD at 415 nm. The percentage of hemolysis was calculated as (OD415_sample_ − OD415_0µM_)/(OD415_TritonX_ − OD415_0µM_) × 100%, where OD415_sample_ is the optical density of the peptide/halicin, OD415_0µM_ is the optical density of the negative control, and OD415_TritonX_ is the optical density of the positive controls. Hemolysis ≤ 5% was considered as safe.

### 4.13. Statistical Analysis 

Statistical analysis was performed using GraphPad Prism software version 6.0 (GraphPad Software, San Diego, CA, USA) and Kruskal–Wallis and Mann–Whitney U tests to determine the significance of the differences between the values for the peptide- and/or halicin-exposed and control samples. All the data were presented as mean; *p* ≤ 0.05 was considered as statistically significant. 

## 5. Conclusions

In conclusion, the present work revealed that the two novel antimicrobial agents, i.e., the synthetic peptide SAAP-148 and halicin, are promising candidates to fight CRIs owing to their high broad-spectrum, antibacterial, anti-biofilm, anti-persister, and anti-inflammatory activities in vitro; but importantly, their ability to act synergistically on biofilm eradication, where their combined effect is superior to their individual effects. This work provides a strong basis for further studies (using a human bladder model and animal models of catheter-related infections and for transcriptomic studies) for the clinical use of SAAP-148 and halicin to treat CRIs caused by Gram-negative bacteria. In addition, the design of more potent and safer therapeutic antimicrobial agents using several strategies, like chemical modification, nanotechnology-based delivery systems, and computer-aided design, is an excellent way to overcome the drawbacks of antimicrobial agents (by reducing their toxicities and improving their antimicrobial activities).

## Figures and Tables

**Table 1:** Characteristics of Gram-negative bacterial strains isolated from intravenous and urinary catheters.

Identity	Origin	* Resistance Profile	** Biofilm Mass (OD Values)	*** Classification [52]	**** Virulence Profile
*Klebsiella pneumoniae* KP1	URC	AMC^R^; FEP^R^; TET^R^; GEN^R^	0.292 ± 0.041 ^⸸⸸^	Strongly adherent	*mrkD+*; *fimH+*; *ycfM+*; *ecpA+*
*Klebsiella pneumoniae* KP2	URC	AMC^R^; CTX^R^; CAZ^R^; FEP^R^; TET^R^; GEN^I^	0.254 ± 0.022 ^⸸⸸^	Strongly adherent	*mrkD+ fimH+*; *ycfM+*; *ecpA+*
*Klebsiella pneumoniae* KP3	URC	AMC^R^; CTX^R^; CAZ^R^; FEP^R^; TET^R^; GEN^R^	0.121 ± 0.031 ^⸸⸸^	Strongly adherent	*mrkD+*; *fimH+*; *ycfM+*; *ecpA+*
*Klebsiella pneumoniae* KP4	URC	AMC^R^; CTX^R^; CAZ^R^; FEP^R^; TET^R^	0.187 ± 0.011 ^⸸⸸^	Strongly adherent	*mrkD+*; *fimH+*; *ycfM+*; *ecpA+*
*Klebsiella pneumoniae* KP5	URC	AMC^R^; CTX^R^; CAZ^R^; FEP^R^; TET^R^; CIP^I^; GEN^I^	0.074 ± 0.031 ^⸸^	Moderately adherent	*mrkD+*; *fimH+*; *ycfM+*; *ecpA+*
*Acinetobacter baumannii* AB1	IVC	CTX^R^; CAZ^R^; FEP^R^; IPM^R^; TET^R^; CIP^R^; CO-TRI^R^	0.242 ± 0.020 ^⸸⸸^	Strongly adherent	*csuE+*; *ompA+*; *bap+*
*Acinetobacter baumannii* AB2	IVC	CTX^R^; CAZ^R^; FEP^R^; IPM^R^; TET^R^; CIP^R^; CO-TRI^R^	0.088 ± 0.013 ^⸸⸸^	Moderately adherent	*csuE+*; *ompA+*; *bap+*
*Acinetobacter baumannii* AB3	IVC	CTX^R^; CAZ^R^; FEP^R^; IPM^R^; TET^R^; CIP^R^; CO-TRI^R^	0.057 ± 0.025 ^⸸^	Weakly adherent	*csuE+*; *ompA+*; *bap+*
*Acinetobacter lwoffii* AL1	IVC	FEP^R^; IPM^R^; TET^R^; CIP^R^; CO-TRI^R^	0.349 ± 0.048 ^⸸⸸^	Strongly adherent	*csuE₋*; *ompA+*; *bap₋*
*Acinetobacter baumannii* AB4	IVC	CTX^R^; CAZ^R^; FEP^R^; IPM^R^; TET^R^; CIP^R^; CO-TRI^R^	0.059 ± 0.159 ^⸸^	Weakly adherent	*csuE+*; *ompA+*; *bap₋*
*Acinetobacter baumannii* AB5	IVC	CTX^R^; CAZ^R^; FEP^R^; IPM^R^; TET^R^; CIP^R^; CO-TRI^R^	0.021 ± 0.013	Non-adherent	*csuE+*; *ompA+*; *bap+*
*Escherichia coli* EC1	IVC	TET^R^; NA^R^	0.03 ± 0.013	Non-adherent	*fimH+*; *hlyF+*; *csgA+*
*Escherichia coli* EC2	URC	TET^R^; GEN^I^; NA^R^	0.376 ± 0.022 ^⸸⸸^	Strongly adherent	*fimH+*; *hlyF₋*; *csgA+*
*Escherichia coli* EC3	IVC	*Sensitive to all antibiotics*	0.028 ± 0.01	Non-adherent	*fimH+*; *hlyF₋*; *csgA+*
*Escherichia coli* EC4	URC	NA^R^	0.101 ± 0.019 ^⸸⸸^	Moderately adherent	*fimH+*; *hlyF₋*; *csgA+*
*Escherichia coli* EC5	URC	CIP^I^; NA^R^	0.259 ± 0.007 ^⸸⸸^	Strongly adherent	*fimH+*; *hlyF+*; *csgA+*
*Escherichia coli* EC6	IVC	NA^R^	0.261 ± 0.032 ^⸸⸸^	Strongly adherent	*fimH+*; *hlyF+*; *csgA+*
*Escherichia coli* EC7	IVC	NA^R^	0249 ± 0.032 ^⸸⸸^	Strongly adherent	*fimH+*; *hlyF₋*; *csgA+*
*Escherichia coli* EC8	IVC	TET^I^; NA^R^	0.292 ± 0.024 ^⸸⸸^	Strongly adherent	*fimH+*; *hlyF+*; *csgA+*
*Escherichia coli* EC9	IVC	TET^R^; NA^R^	0.222 ± 0.012 ^⸸⸸^	Strongly adherent	*fimH+*; *hlyF+*; *csgA+*
*Escherichia coli* EC10	IVC	AMC^R^; NA^R^	0.284 ± 0.018 ^⸸⸸^	Strongly adherent	*fimH+*; *hlyF-*; *csgA+*
*Escherichia coli* EC11	IVC	AMC^R^; CO-TRI^R^	0.027 ± 0.011	Non-adherent	*fimH+*; *hlyF₋*; *csgA+*
*Escherichia coli* EC12	URC	NA^R^	0.068 ± 0.022 ^⸸^	Moderately adherent	*fimH+*; *hlyF₋*; *csgA+*
*Escherichia coli* EC13	IVC	CIP^I^	0.243 ± 0.015 ^⸸⸸^	Strongly adherent	*fimH+*; *hlyF₋*; *csgA+*
*Escherichia coli* EC14	IVC	AMC^R^; CO-TRI^R^	0.051 ± 0.007 ^⸸^	Weakly adherent	*fimH+*; *hlyF₋*; *csgA+*
*Escherichia coli* EC15	IVC	AMC^R^; TET^R^	0.044 ± 0.019 ^ns^	Weakly adherent	*fimH+*; *hlyF+*; *csgA+*
*Escherichia coli* EC16	IVC	TET^R^; NA^R^	0.094 ± 0.008 ^⸸⸸^	Moderately adherent	*fimH+*; *hlyF₋*; *csgA+*
*Escherichia coli* EC17	IVC	TET^R^; NA^R^	0.141 ± 0.017 ^⸸⸸^	Strongly adherent	*fimH+*; *hlyF₋*; *csgA+*
*Escherichia coli* EC18	IVC	CIP^I^; NA^R^	0.096 ± 0.011 ^⸸⸸^	Moderately adherent	*fimH+*; *hlyF+*; *csgA+*
*Escherichia coli* EC19	IVC	TET^R^; NA^R^	0.047 ± 0.034 ^ns^	Weakly adherent	*fimH+*; *hlyF₋*; *csgA+*
*Escherichia coli* EC20	IVC	CIP^I^; NA^R^	0.131 ± 0.032 ^⸸⸸^	Strongly adherent	*fimH+*; *hlyF+*; *csgA+*
*Escherichia coli* EC21	IVC	TET^R^; NA^R^	0.04 ± 0.023 ^ns^	Weakly adherent	*fimH+*; *hlyF+*; *csgA+*
*Escherichia coli* EC22	IVC	AMC^R^; NA^R^	0.122 ± 0.003 ^⸸⸸^	Strongly adherent	*fimH+*; *hlyF+*; *csgA+*
*Escherichia coli* EC23	IVC	CIP^I^; NA^R^	0.105 ± 0.007 ^⸸⸸^	Moderately adherent	*fimH+*; *hlyF+*; *csgA+*
*Escherichia coli* EC24	IVC	TET^R^; NA^R^	0.101 ± 0.029 ^⸸⸸^	Moderately adherent	*fimH+*; *hlyF+*; *csgA+*
*Escherichia coli* EC25	IVC	TET^R^; NA^R^	0.042 ± 0.015 ^ns^	Weakly adherent	*fimH+*; *hlyF+*; *csgA+*
*Escherichia coli* EC26	IVC	AMC^R^; NA^R^	0.039 ± 0.006 ^ns^	Weakly adherent	*fimH+*; *hlyF+*; *csgA+*
*Escherichia coli* EC27	IVC	TET^R^; NA^R^	0.024 ± 0.003	Non-adherent	*fimH+*; *hlyF+*; *csgA+*
*Escherichia coli* EC28	IVC	AMC^R^; CO-TRI^R^	0.023 ± 0.011	Non-adherent	*fimH+*; *hlyF₋*; *csgA+*
*Escherichia coli* EC29	IVC	*Sensitive to all antibiotics*	0.052 ± 0.008 ^⸸^	Weakly adherent	*fimH+*; *hlyF₋*; *csgA₋*

IVC: intravenous catheter; URC: urinary catheter; bacteria were coded “EC” for *Escherichia coli*, “AB” for *Acinetobacter baumannii*, and “KP” for *Klebsiella pneumoniae*. *Only intermediate (I)/resistance(R) is indicated. Tested antibiotics: AMC: amoxicillin + clavulanic acid; CTX: cefotaxime; CAZ: ceftazidime; FOX: cefoxitin; ERY: erythromycin; TET: tetracycline; CIP: ciprofloxacin; GEN: gentamycin; IMP: imipenem; FEP: cefepime; NA: nalidixic acid; CO-TRI: cotrimoxazole. ** Biofilm mass; n = 3 independent experiments each in triplicate. Mann–Whitney U test: ^⸸^ *p* < 0.05 indicates that OD_sample_ is significantly different from OD_control_; ns: not significantly different. OD_control_ = 0.03. *** The strains were classified as follows: non-adherent (OD_sample_ ≤ 0.03); weakly adherent (0.03 < OD_sample_ ≤ 0.06); moderately adherent (0.06 < OD_sample_ ≤ 0.12); strongly adherent (OD_sample_ > 0.12). **** Virulence genes; +: gene present; ₋: gene absent.

**Table 2:** Bactericidal efficacies of SAAP-148 peptide and halicin against antibiotic-resistant Gram-negative strains isolated from catheters.

Strains	Antibiotic Resistances											LC 99.9% of SAAP-148 (µM)				LC 99.9% of Halicin (µM)			
												4 h		24 h		4 h		24 h	
	AMC	CTX	CAZ	FEP	FOX	IPM	TET	CIP	GEN	NA	CO-TRI	PBS	50% Plasma or Urine	PBS	50% Plasma or Urine	PBS	50% Plasma or Urine	PBS	50% Plasma or Urine
*E. coli* EC2												0.8	3.2	0.8	12.8	25.6 (12.8–25.6)	102.4 (51.2–102.4)	6.4	25.6 (12.8–25.6)
*A. baumannii* AB1										/		0.8	1.6	0.8	3.2	25.6	>102.4	25.6	>102.4
*K. pneumoniae* KP1												0.8 (0.8–1.6)	6.4	1.6	12.8	51.2 (25.6–51.2)	25.6	51.2	51.2
*K. pneumoniae* KP2												1.6 (0.8–1.6)	6.4 (3.2–6.4)	1.6	12.8	25.6 (12.8–25.6)	51.2 (25.6–51.2)	25.6	102.4 (51.2–102.4)

An in vitro killing assay was used to determine the bactericidal activities of SAAP-148 and halicin when administered in PBS; 50% urine (EC2, KP1, and KP2); and 50% human plasma (AB1). Approximately 2% (*v*/*v*) of the bacteria were mixed with increasing concentrations of SAAP-148 or halicin and after 4 h and 24 h, the number of surviving bacteria was assessed microbiologically. The results are expressed as LC 99.9% values, i.e., the lowest concentration of the antimicrobial agent that results in a 99.9% reduction in the bacterial count compared to that of the control. The values are the means (and ranges) of six replicates from three independent experiments. If no range is mentioned, the LC 99.9% was identical in all the experiments. AMC: amoxicillin + clavulanic acid; CTX: cefotaxime; CAZ: ceftazidime; FEP: cefepime; FOX: cefoxitin; IMP: imipenem; TET: tetracycline; CIP: ciprofloxacin; GEN: gentamycin; NA: nalidixic acid; CO-TRI: cotrimoxazole; sensitive: green box; resistant/intermediate: red box; not tested: white box.

**Table 3:** Synergistic effects of SAAP-148 and halicin alone/in combination against GNB preformed biofilms and fractional biofilm eradication concentration indexes (ΣFBEC) for combinations of SAAP-148 and halicin.

Strain	MBEC SAAP-148 (µM) Alone	MBEC SAAP-148 (µM) in Combination	MBEC Halicin (µM) Alone	MBEC Halicin (µM) in Combination	ΣFBEC
*E. coli* EC2	102.4	3.2	25.6	6.4	0.28 (Synergistic effect)
		3.2		12.8	0.53 (Additive effect)
		6.4		12.8	0.56 (Additive effect)
		12.8		6.4	0.38 (Synergistic effect)
		12.8		12.8	0.63 (Additive effect)
		25.6		6.4	0.5 (Synergistic effect)
		25.6		12.8	0.75 (Additive effect)
*A. baumannii* AB1	102.4	No effect	102.4	No effect	No effect
*K. pneumoniae* KP1	51.2	12.8	102.4	12.8	0.38 (Synergistic effect)
		12.8		25.6	0.5 (Synergistic effect)
*K. pneumoniae* KP2	51.2	3.2	102.4	102.4	1.06 (Indifferent effect)
		12.8		12.8	0.38 (Synergistic effect)
		12.8		25.6	0.5 (Synergistic effect)
		12.8		51.2	0.75 (Additive effect)
		12.8		102.4	1.25 (Indifferent effect)
		25.6		51.2	1 (Additive effect)
		25.6		102.4	1.5 (Indifferent effect)

In short, 24 h biofilms on sterile silicone elastomer discs were washed twice and then exposed to increasing concentrations of SAAP-148 and halicin (alone) and to different concentrations of SAAP-148 and halicin (in combination) for 24 h at 37 °C. Thereafter, the discs were washed twice after the gentle removal of the antimicrobial agents, and the remaining biofilms were sonicated for 10 min at 42 kHz. The number of surviving bacteria was determined microbiologically. The experiment was assessed four times each in duplicate. MBEC: minimum biofilm eradication concentration. Synergy is defined by the fractional biofilm eradication concentration index (ΣFBEC) ≤ 0.5; additive effect as 0.5 < ΣFBEC ≤ 1; indifference as 1 < ΣFBEC ≤ 2; antagonism as ΣFBEC > 2.

## Supplementary Materials

The following supporting information can be downloaded at https://www.mdpi.com/article/10.3390/antibiotics12121743/s1: Table S1: Antibiotics tested in this study; Table S2: Target genes and primers used in this study; Table S3: PCR programs for virulence gene detection; Table S4: Concentrations of SAAP-148 and halicin tested in checkerboard assays. References [139,140,141,142,143,144,145,146,147] are cited in the Supplementary Materials.

## References

[1] Patel A.R., Patel A.R., Singh S., Singh S., Khawaja I. (2019). Central line catheters and associated complications: A review. Cureus.

[2] Jatczak L., Puton R.C., Proença A.J.L., Rubin L.C., Borges L.B., Saleh J.N., Corrêa M.P. (2023). Complications of central venous catheterization at a vascular surgery service in a teaching hospital: A prospective cohort study. J. Vasc. Bras..

[3] VanEpps J.S., Younger J.G. (2016). Implantable device-related infection. Shock.

[4] Dadi N.C.T., Radochová B., Vargová J., Bujdáková H. (2021). Impact of healthcare-associated infections connected to medical devices—An update. Microorganisms.

[5] Drugeon B., Guenezan J., Pichon M., Devos A., Fouassin X., Neveu A., Boinot L., Pratt V., Mimoz O. (2023). Incidence, complications, and costs of peripheral venous catheter-related bacteraemia: A retrospective, single-centre study. J. Hosp. Infect..

[6] Voidazan S., Albu S., Toth R., Grigorescu B., Rachita A., Moldovan I. (2020). Healthcare associated infections—A new pathology in medical practice?. Int. J. Environ. Res. Public Health.

[7] Gahlot R., Nigam C., Kumar V., Yadav G., Anupurba S. (2014). Catheter-related bloodstream infections. Int. J. Crit. Illn. Inj. Sci..

[8] Feneley R.C., Hopley I.B., Wells P.N. (2015). Urinary catheters: History, current status, adverse events and research agenda. J. Med. Eng. Technol..

[9] Zhang S., Wang L., Liang X., Vorstius J., Keatch R., Corner G., Nabi G., Davidson F., Gadd G.M., Zhao Q. (2019). Enhanced antibacterial and antiadhesive activities of silver-PTFE nanocomposite coating for urinary catheters. ACS. Biomater. Sci. Eng..

[10] Khatoon Z., McTiernan C.D., Suuronen E.J., Mah T.F., Alarcon E.I. (2018). Bacterial biofilm formation on implantable devices and approaches to its treatment and prevention. Heliyon.

[11] Solis-Velazquez O.A., Gutiérrez-Lomelí M., Guerreo-Medina P.J., de Lourdes Rosas-García M., Iñiguez-Moreno M., Avila-Novoa M.G. (2021). Nosocomial pathogen biofilms on biomaterials: Different growth medium conditions and components of biofilms produced in vitro. J. Microbiol. Immunol. Infect..

[12] Thorarinsdottir H.R., Rockholt M., Klarin B., Broman M., Fraenkel C.J., Kander T. (2020). Catheter-related infections: A Scandinavian observational study on the impact of a simple hygiene insertion bundle. Acta Anaesthesiol. Scand..

[13] Ripa M., Morata L., Rodríguez-Núñez O., Cardozo C., Puerta-Alcalde P., Hernández-Meneses M., Ambrosioni J., Linares L., Bodro M., Valcárcel A. (2018). Short-term peripheral venous catheter-related bloodstream infections: Evidence for increasing prevalence of Gram-negative microorganisms from a 25-Year prospective observational study. Antimicrob. Agents Chemother..

[14] Tsuboi M., Hayakawa K., Mezaki K., Katanami Y., Yamamoto K., Kutsuna S., Takeshita N., Ohmagari N. (2019). Comparison of the epidemiology and microbiology of peripheral line-and central line associated bloodstream infections. Am. J. Infect. Control.

[15] Surapat B., Montakantikul P., Malathum K., Kiertiburanakul S., Santanirand P., Chindavijak B. (2020). Microbial epidemiology and risk factors for relapse in Gram-negative bacteria catheter-related bloodstream infection with a pilot prospective study in patients with catheter removal receiving short-duration of antibiotic therapy. BMC. Infect. Dis..

[16] Lendak D., Puerta-Alcalde P., Moreno-García E., Chumbita M., García-Pouton N., Cardozo C., Morata L., Suárez-Lledó M., Hernández-Meneses M., Ghiglione L. (2021). Changing epidemiology of catheter-related bloodstream infections in neutropenic oncohematological patients. PLoS ONE.

[17] Mandolfo S., Anesi A., Rognoni V. (2022). The epidemiology of central venous catheter-related bloodstream infection in our renal units is changing. J. Vasc. Access.

[18] Neoh K.G., Li M., Kang E.T., Chiong E., Tambyah P.A. (2017). Surface modification strategies for combating catheter-related complications: Recent advances and challenges. J. Mater. Chem. B.

[19] Felix L., Whitely C., Tharmalingam N., Mishra B., Vera-Gonzalez N., Mylonakis E., Shukla A., Fuchs B.B. (2023). Auranofin coated catheters inhibit bacterial and fungal biofilms in a murine subcutaneous model. Front. Cell. Infect. Microbiol..

[20] Bassetti M., Montero J.G., Paiva J.A. (2017). When antibiotic treatment fails. Intensive Care Med..

[21] Köves B., Magyar A., Tenke P. (2017). Spectrum and antibiotic resistance of catheter-associated urinary tract infections. GMS. Infect. Dis..

[22] Cepas V., López Y., Muñoz E., Rolo D., Ardanuy C., Martí S., Xercavins M., Horcajada J.P., Bosch J., Soto S.M. (2018). Relationship between biofilm formation and antimicrobial resistance in Gram-Negative Bacteria. Microb. Drug Resist..

[23] Windels E.M., Michiels J.E., Van den Bergh B., Fauvart M., Michiels J. (2019). Antibiotics: Combatting tolerance to stop resistance. mBio.

[24] Di Domenico E.G., Oliva A., Guembe M. (2022). The current knowledge on the pathogenesis of tissue and medical device-related biofilm infections. Microorganisms.

[25] Chernysh S., Gordya N., Tulin D., Yakovlev A. (2018). Biofilm infections between Scylla and Charybdis: Interplay of host antimicrobial peptides and antibiotics. Infect. Drug Resist..

[26] Grassi L., Maisetta G., Esin S., Batoni G. (2017). Combination strategies to enhance the efficacy of antimicrobial peptides against bacterial biofilms. Front. Microbiol..

[27] Zapotoczna M., Forde É., Hogan S., Humphreys H., O’Gara J.P., Fitzgerald-Hughes D., Devocelle M., O’Neill E. (2017). Eradication of *Staphylococcus aureus* biofilm infections using synthetic antimicrobial peptides. J. Infect. Dis..

[28] de Breij A., Riool M., Cordfunke R.A., Malanovic N., de Boer L., Koning R.I., Ravensbergen E., Franken M., Van der Heijde T., Boekema B.K. (2018). The antimicrobial peptide SAAP-148 combats drug-resistant bacteria and biofilms. Sci. Transl. Med..

[29] Van der Does A.M., Hiemstra P.S., Mookherjee N. (2019). Antimicrobial host defense peptides: Immunomodulatory functions and translational prospects. Adv. Exp. Med. Biol..

[30] Kim Y.M., Son H.P.S.C., Lee J.K., Jang M.K., Lee J.R. (2023). Anti-biofilm effects of rationally designed peptides against planktonic cells and pre-formed Biofilm of *Pseudomonas aeruginosa*. Antibiotics.

[31] di Luca M., Maccari G., Nifosì R. (2014). Treatment of microbial biofilms in the post-antibiotic era: Prophylactic and therapeutic use of antimicrobial peptides and their design by bioinformatics tools. Pathog. Dis..

[32] Jaśkiewicz M., Neubauer D., Kazor K., Bartoszewska S., Kamysz W. (2018). Antimicrobial activity of selected antimicrobial peptides against planktonic culture and biofilm of *Acinetobacter baumannii*. Probiotics Antimicrob. Proteins.

[33] Feng X., Sambanthamoorthy K., Palys T., Paranavitana C. (2013). The human antimicrobial peptide LL-37 and its fragments possess both antimicrobial and antibiofilm activities against multidrug-resistant *Acinetobacter baumannii*. Peptides.

[34] Luo Y., McLean D.T.F., Linden G.J., McAuley D.F., McMullan R., Lundy F.T. (2017). The naturally occurring host defenses peptide, LL-37, and its truncated mimetics KE-18 and KR-12 have selected biocidal and antibiofilm activities against *Candida albicans*, *Staphylococcus aureus*, and *Escherichia coli in vitro*. Front. Microbiol..

[35] Spencer J.J., Pitts R.E., Pearson R.A., King L.B. (2018). The effects of antimicrobial peptides WAM-1 and LL-37 on multidrug-resistant *Acinetobacter baumannii*. Pathog. Dis..

[36] van Gent M.E., van der Reijden T.J.K., Lennard P.R., de Visser A.W., Schonkeren-Ravensbergen B., Dolezal N., Cordfunke R.A., Drijfhout J.W., Nibbering P.H. (2022). Synergism between the synthetic antibacterial and antibiofilm peptide (SAAP)-148 and halicin. Antibiotics.

[37] Scheper H., Wubbolts J.M., Verhagen J.A.M., de Visser A.W., van der Wall R.J.P., Visser L.G., de Boer M.G.J., Nibbering P.H. (2020). SAAP-148 eradicates MRSA persisters within mature biofilm models simulating prosthetic joint infection. Front. Microbiol..

[38] Verheul M., Drijfhout J.W., Pijls B.G., Nibbering P.H. (2021). Non-contact induction heating and SAAP-148 eliminate persisters within MRSA biofilms mimicking a metal implant infection. Eur. Cells. Mater..

[39] Stokes J.M., Yang K., Swanson K., Jin W., Cubillos-Ruiz A., Donghia N.M., MacNair C.R., French S., Carfrae L.A., Bloom-Ackermann Z. (2020). A deep learning approach to antibiotic discovery. Cell.

[40] Booq R.Y., Tawfik E.A., Alfassam H.A., Alfahad A.J., Alyamani E.J. (2021). Assessment of the antibacterial efficacy of halicin against pathogenic bacteria. Antibiotics.

[41] Aburayan W.S., Booq R.Y., BinSaleh N.S., Alfassam H.A., Bakr A.A., Bukhary H.A., Alyamani E.J., Tawfik E.A. (2020). The delivery of the novel drug ‘Halicin’ using electrospun fibers for the treatment of pressure ulcer against pathogenic bacteria. Pharmaceutics.

[42] Koppen B.C., Mulder P.P.G., de Boer L., Riool M., Drijfhout J.W., Zaat S.A.J. (2019). Synergistic microbicidal effect of cationic antimicrobial peptides and teicoplanin against planktonic and biofilm-encased *Staphylococcus aureus*. Int. J. Antimicrob. Agents.

[43] Dijksteel G.S., Ulrich M.M.W., Vlig M., Nibbering P.H., Cordfunke R.A., Drijfhout J.W., Middelkoop E., Boekema B.K.H.L. (2019). Potential factors contributing to the poor antimicrobial efficacy of SAAP-148 in a rat wound infection model. Ann. Clin. Microbiol. Antimicrob..

[44] Li H., Xu L., Liu Y., Pengfei S., Yong W.U. (2021). Antibacterial effects of small molecule antidiabetic agent Halicin against *Staphylococcus aureus*. Chin. J. Lab. Med..

[45] Higashihira S., Simpson S.J., Collier C.D., Natoli R.M., Kittaka M., Greenfield E.M. (2022). Halicin is effective against *Staphylococcus aureus* biofilms in vitro. Clin. Orthop. Relat. Res..

[46] Hussain Z., Pengfei S., Yimin L., Shasha L., Zehao L., Yifan Y., Linhui L., Linying Z., Yong W. (2022). Study on antibacterial effect of halicin (SU3327) against *Enterococcus faecalis* and *Enterococcus faecium*. Pathog. Dis..

[47] Van Gent M.E., Schonkeren-Ravensbergen B., Achkif A., Beentjes D., Dolezal N., Van Meijgaarden K.E., Drijfhout J.W., Nibbering P.H. (2023). C-terminal PEGylation improves SAAP-148 peptide’s immunomodulatory activities. J. Innate Immun..

[48] van Gent M.E., Klodzinska S.N., Wouter Drijfhout J., Nielsen H.M., Nibbering P.H. (2023). Encapsulation in oleyl-modified hyaluronic acid nanogels substantially improves the clinical potential of the antimicrobial peptides SAAP-148 and Ab-Cath. Eur. J. Pharm. Biopharm..

[49] Ali M., van Gent M.E., de Waal A.M., van Doodewaerd B.R., Bos E., Koning R.I., Cordfunke R.A., Drijfhout J.W., Nibbering P.H. (2023). Physical and functional characterization of PLGA nanoparticles containing the antimicrobial peptide SAAP-148. Int. J. Mol. Sci..

[50] Schmitz M.G.J., Riool M., de Boer L., Vrehen A.F., Bartels P.A.A., Zaat S.A.J., Dankers P.Y.W. (2023). Development of an antimicrobial peptide SAAP-148-functionalized supramolecular coating on Titanium to prevent biomaterial-associated infections. Adv. Mater. Technol..

[51] Ön A., Vejzovic D., Jennings J., Parigger L., Cordfunke R.A., Drijfhout J.W., Lohner K., Malanovic N. (2023). Bactericidal activity to *Escherichia coli*: Different modes of action of two 24-Mer peptides SAAP-148 and OP-145, both derived from human Cathelicidine LL-37. Antibiotics.

[52] Stepanovic S., Vukovic D., Dakic I., Savic B., Svabic-Vlahovic M. (2000). A modified microtiter-plate test for quantification of staphylococcal biofilm formation. J. Microbiol. Methods.

[53] Buetti N., Lo Priore E., Atkinson A., Widmer A.F., Kronenberg A., Marschall J., Swiss Centre for Antibiotic Resistance (ANRESIS) (2018). Catheter-related infections: Does the spectrum of microbial causes change over time? A nationwide surveillance study. BMJ Open.

[54] Namiganda V., Mina Y., Meklat A., Touati D., Bouras N., Barakate M., Sabaou N. (2019). Antibiotic Resistance Pattern of *Acinetobacter baumannii* Strains Isolated from Different Clinical Specimens and Their Sensibility Against Bioactive Molecules Produced by *Actinobacteria*. Arab. J. Sci. Eng..

[55] Benamrouche N., Lafer O., Benmahdi L., Benslimani A., Amhis W., Houria Ammari H., Assaous F., Azzam A., Rahal K., Tali Maamar H.J. (2020). Phenotypic and genotypic characterization of multidrug-resistant *Acinetobacter baumannii* isolated in Algerian hospitals. Infect. Dev. Ctries.

[56] Benzaid C., Tichati L., Rouabhia M., Akil Dahdouh S. (2022). Prevalence of microbial nosocomial infections in the resuscitation unit of the University Hospital of Annaba-Algeria. Ann. Biol. Clin..

[57] Harzallah B., Grama B.S., Benabdelmalek A., Mekhloufi I. (2022). Incidence of nosocomial infections in reanimation unit at the hospital of Constantine (Algeria). South Asian J. Exp. Biol..

[58] Boulesnam S.L., Hamaidi-Chergui F., Benamara M., Azrou S. (2023). Phenotypical Comparison between Environmental and Clinical *Acinetobacter baumannii* Strains Isolated from an Intensive Care Unit. Malays. J. Med. Sci..

[59] Strasheim W., Kock M.M., Ueckermann V., Hoosien E., Dreyer A.W., Ehlers M.M. (2015). Surveillance of catheter-related infections: The supplementary role of the microbiology laboratory. BMC Infect. Dis..

[60] Navab-Daneshmand T., Friedrich M.N.D., Gächter M., Montealegre M.C., Mlambo L.S., Nhiwatiwa T., Mosler H.J., Julian T.R. (2018). *Escherichia coli* Contamination across Multiple Environmental Compartments (Soil, Hands, Drinking Water, and Handwashing Water) in Urban Harare: Correlations and Risk Factors. Am. J. Trop. Med. Hyg..

[61] Daga A.P., Koga V.L., Soncini J.G.M., de Matos C.M., Perugini M.R.E., Pelisson M., Kobayashi R.K.T., Vespero E.C. (2019). *Escherichia coli* Bloodstream Infections in Patients at a University Hospital: Virulence Factors and Clinical Characteristics. Front. Cell. Infect. Microbiol..

[62] Barbadoro P., Labricciosa F.M., Recanatini C., Gori G., Tirabassi F., Martini E., Gioia M.G., D’Errico M.M., Prospero E. (2015). Catheter-associated urinary tract infection: Role of the setting of catheter insertion. Am. J. Infect. Control.

[63] Weiner L., Webb A., Limbago B., Dudeck M., Patel J., Kallen A., Edwards J.R., Sievert D. (2016). Antimicrobial-resistant pathogens associated with healthcare-associated infections: Summary of data reported to the National Healthcare Safety Network at the Centers for Disease Control and Prevention, 2011–2014. Infect. Control Hosp. Epidemiol..

[64] Dybowski B.A., Zapała P., Bres-Niewada E.Z.L., Miązek-Zapała N., Poletajew S., Młynarczyk G., Radziszewski P. (2018). Catheter-associated bacterial flora in patients with benign prostatic hyperplasia: Shift in antimicrobial susceptibility pattern. BMC Infect. Dis..

[65] Peng D., Li X., Liu P., Luo M., Chen S., Su K., Zhang Z., He Q., Qiu J., Li Y. (2018). Epidemiology of pathogens and antimicrobial resistance of catheter-associated urinary tract infections in intensive care units: A systematic review and meta-analysis. Am. J. Infect. Control.

[66] Aouf A., Gueddi T., Djeghout B., Ammari H. (2018). Frequency and susceptibility pattern of uropathogenic *Enterobacteriaceae* isolated from patients in Algiers, Algeria. J. Infect. Dev. Ctries.

[67] Nabti L.Z., Sahli F., Radji N., Mezaghcha W., Semara L., Aberkane S., Lounnas M., Solassol J., Didelot M.N., Jean-Pierre H. (2019). High prevalence of multidrug-resistant *Escherichia coli* in urine samples from inpatients and outpatients at a tertiary care Hospital in Setif, Algeria. Microb. Drug. Resist..

[68] Ait-Mimoune N., Hassaine H., Boulanoir M. (2022). Bacteriological profile of urinary tract infections and antibiotic susceptibility of *Escherichia coli* in Algeria. Iran. J. Microbiol..

[69] Flores-Mireles A., Hreha T.N., Hunstad D.A. (2019). Pathophysiology, treatment and prevention of catheter-associated urinary tract infection. Top. Spinal. Cord. Inj. Rehabil..

[70] Alcántar-Curiel M.D., Ledezma-Escalante C.A., Jarillo-Quijada M.A., Gayosso-Vázquez C., Morfín-Otero R., Rodríguez-Noriega E., Cedillo-Ramírez M.L., Santos-Preciado J.I., Girón J.A. (2018). Association of antibiotic resistance, cell adherence, and biofilm production with the endemicity of nosocomial *Klebsiella pneumoniae*. BioMed. Res. Int..

[71] Ramos-Vivas J., Chapartegui-González I., Fernández-Martínez M., González-Rico C., Fortún J., Escudero R., Marco F., Linares L., Montejo M., Aranzamendi M. (2019). Biofilm formation by multidrug resistant *Enterobacteriaceae* strains isolated from solid organ transplant recipients. Sci. Rep..

[72] Lin M.F., Lin Y.Y., Lan C.Y. (2020). Characterization of biofilm production in different strains of *Acinetobacter baumannii* and the effects of chemical compounds on biofilm formation. PeerJ.

[73] Pontes C., Alves M., Santos C., Ribeiro M.H., Gonçalves L., Bettencourt A.F., Ribeiro I.A.C. (2016). Can Sophorolipids prevent biofilm formation on silicone catheter tubes?. Int. J. Pharm..

[74] Azam M.W., Zuberi A., Khan A.U. (2020). *bolA* gene involved in curli amyloids and fimbriae production in *E. coli*: Exploring pathways to inhibit biofilm and amyloid formation. J. Biol. Res..

[75] Colquhoun J.M., Rather P.N. (2020). Insights into mechanisms of biofilm formation in *Acinetobacter baumannii* and implications for Uropathogenesis. Front. Cell. Infect. Microbiol..

[76] Nie D., Hu Y., Chen Z., Li M., Hou Z., Luo X., Mao X., Xue X. (2020). Outer membrane protein A (OmpA) as a potential therapeutic target for *Acinetobacter baumannii* infection. J. Biomed. Sci..

[77] Pompilio A., Scribano D., Sarshar M., Di Bonaventura G., Palamara A.T., Ambrosi C. (2021). Gram-negative bacteria holding together in a biofilm: The *Acinetobacter baumannii* way. Microorganisms.

[78] Ferreira R.L., da Silva B., Rezende G.S., Nakamura-Silva R., Pitondo-Silva A., Campanini E.B., Brito M., da Silva E., Freire C., da Cunha A.F. (2019). High prevalence of multidrug-resistant *Klebsiella pneumoniae* harboring several virulence and β-Lactamase encoding genes in a Brazilian intensive care unit. Front. Microbiol..

[79] Ayad A., Drissi M., de Curraize C., Dupont C., Hartmann A., Solanas S., Siebor E., Amoureux L., Neuwirth C. (2016). Occurence of *ArmA* and *RmtB* aminoglycoside resistance 16S rRNA methylases in extended-spectrum β-Lactamases producing *Escherichia coli* in Algerian Hospitals. Front. Microbiol..

[80] Bourafa N., Chaalal W., Bakour S., Lalaoui R., Boutefnouchet N., Diene S.M., Rolain J.M. (2018). Molecular characterization of carbapenem-resistant Gram-negative bacilli clinical isolates in Algeria. Infect. Drug Resist..

[81] Zenati F., Barguigua A., Nayme K., Benbelaïd F., Khadir A., Bellahsene C., Bendahou M., Hassaine H., Timinouni M. (2019). Characterization of uropathogenic ESBL-producing *Escherichia coli* isolated from hospitalized patients in western Algeria. J. Infect. Dev. Ctries.

[82] Shenkutie A.M., Yao M.Z., Siu G.K., Wong B., Leung P.H. (2020). Biofilm-Induced Antibiotic Resistance in Clinical *Acinetobacter baumannii* Isolates. Antibiotics.

[83] Olivares E., Badel-Berchoux S., Provot C., Prévost G., Bernardi T., Jehl F. (2020). Clinical Impact of Antibiotics for the Treatment of *Pseudomonas aeruginosa* Biofilm Infections. Front. Microbiol..

[84] Topka-Bielecka G., Dydecka A., Necel A., Bloch S., Nejman-Faleńczyk B., Węgrzyn G., Węgrzyn A. (2021). Bacteriophage-Derived Depolymerases against Bacterial Biofilm. Antibiotics.

[85] Zhao A., Sun J., Liu Y. (2023). Understanding bacterial biofilms: From definition to treatment strategies. Front. Cell. Infect. Microbiol..

[86] Alves M.J., Barreira J.C., Carvalho I., Trinta L., Perreira L., Ferreira I.C.F.R., Pintado M. (2014). Propensity for biofilm formation by clinical isolates from urinary tract infections: Developing a multifactorial predictive model to improve antibiotherapy. J. Med. Microbiol..

[87] Poursina F., Sepehrpour S., Mobasherizadeh S. (2018). Biofilm Formation in Nonmultidrug-resistant *Escherichia coli* Isolated from Patients with Urinary Tract Infection in Isfahan, Iran. Adv. Biomed. Res..

[88] Osthoff M., McGuinness S.L., Wagen A.Z., Eisen D.P. (2015). Urinary tract infections due to extended-spectrum beta-lactamase-producing Gram-negative bacteria: Identification of risk factors and outcome predictors in an Australian tertiary referral hospital. Int. J. Infect. Dis..

[89] Ranjbar R., Fatahian Kelishadrokhi A., Chehelgerdi M. (2019). Molecular characterization, serotypes and phenotypic and genotypic evaluation of antibiotic resistance of the *Klebsiella pneumoniae* strains isolated from different types of hospital-acquired infections. Infect. Drug Resist..

[90] Caneiras C., Lito L., Melo-Cristino J., Duarte A. (2019). Community- and hospital-acquired *Klebsiella pneumoniae* urinary tract infections in Portugal: Virulence and antibiotic resistance. Microorganisms.

[91] Cusumano J.A., Caffrey A.R., Daffinee K.E., Luther M.K., Lopes V., LaPlante K.L. (2019). Weak biofilm formation among carbapenem-resistant *Klebsiella pneumoniae*. Diagn. Microbiol. Infect. Dis..

[92] Khorsi K., Messai Y., Hamidi M., Ammari H., Bakour R. (2015). High prevalence of multidrug-resistance in *Acinetobacter baumannii* and dissemination of carbapenemase-encoding genes blaOXA-23-like, blaOXA-24-like and blaNDM-1 in Algiers hospitals. Asian Pac. J. Trop. Med..

[93] Bakour S., Olaitan A.O., Ammari H., Touati A., Saoudi S., Saoudi K., Rolain J.M. (2015). Emergence of Colistin- and Carbapenem-resistant *Acinetobacter baumannii* ST2 clinical isolate in Algeria: First Case Report. Microb. Drug Resist..

[94] de Breij A., Riool M., Kwakman P.H., de Boer L., Cordfunke R.A., Drijfhout J.W., Cohen O., Emanuel N., Zaat S.A., Nibbering P.H. (2016). Prevention of *Staphylococcus aureus* biomaterial-associated infections using a polymer-lipid coating containing the antimicrobial peptide OP-145. J. Control. Release.

[95] Nussbaumer-Pröll A., Zeitlinger M. (2020). Use of Supplemented or Human Material to Simulate PD Behavior of Antibiotics at the Target Site *In vitro*. Pharmaceutics.

[96] Wu K.C., Hua K.F., Yu Y.H., Cheng Y.H., Cheng T.T., Huang Y.K., Chang H.W., Chen W.J. (2021). Antibacterial and Antibiofilm Activities of Novel Antimicrobial Peptides against Multidrug-Resistant Enterotoxigenic *Escherichia Coli*. Int. J. Mol. Sci..

[97] Verderosa A.D., Totsika M., Fairfull-Smith K.E. (2019). Bacterial Biofilm Eradication Agents: A Current Review. Front. Chem..

[98] Soares A., Roussel V., Pestel-Caron M., Barreau M., Caron F., Bouffartigues E., Chevalier S., Etienne M. (2019). Understanding Ciprofloxacin Failure in *Pseudomonas aeruginosa* Biofilm: Persister Cells Survive Matrix Disruption. Front. Microbiol..

[99] Wang L., Di Luca M., Tkhilaishvili T., Trampuz A., Gonzalez Moreno M. (2019). Synergistic Activity of Fosfomycin, Ciprofloxacin, and Gentamicin against *Escherichia coli* and *Pseudomonas aeruginosa* Biofilms. Front. Microbiol..

[100] Vestby L.K., Grønseth T., Simm R., Nesse L.L. (2020). Bacterial biofilm and its role in the pathogenesis of disease. Antibiotics.

[101] Bi Y., Xia G., Shi C., Wan J., Liu L., Chen Y., Wu Y., Zhang W., Zhou M., He H. (2021). Therapeutic strategies against bacterial biofilms. Fundam. Res..

[102] Jang M., Kim J., Choi Y., Bang J., Kim Y. (2019). Antiseptic Effect of Ps-K18: Mechanism of Its Antibacterial and Anti-Inflammatory Activities. Int. J. Mol. Sci..

[103] Gan B.H., Gaynord J., Rowe S.M., Deingruber T., Spring D.R. (2021). The multifaceted nature of antimicrobial peptides: Current synthetic chemistry approaches and future directions. Chem. Soc. Rev..

[104] Heinbockel L., Weindl G., Martinez-de-Tejada G., Correa W., Sanchez-Gomez S., Bárcena-Varela S., Goldmann T., Garidel P., Gutsmann T., Brandenburg K. (2018). Inhibition of Lipopolysaccharide- and Lipoprotein-Induced Inflammation by Antitoxin Peptide Pep19-2.5. Front. Immunol..

[105] Ridyard K.E., Overhage J. (2021). The Potential of Human Peptide LL-37 as an Antimicrobial and Anti-Biofilm Agent. Antibiotics.

[106] Krishnan M., Choi J., Choi S., Kim Y. (2021). Anti-Endotoxin 9-Meric Peptide with Therapeutic Potential for the Treatment of Endotoxemia. J. Microbiol. Biotechnol..

[107] Luo Y., Song Y. (2021). Mechanism of Antimicrobial Peptides: Antimicrobial, Anti-Inflammatory and Antibiofilm Activities. Int. J. Mol. Sci..

[108] Ebbensgaard A., Mordhorst H., Overgaard M.T., Aarestrup F.M., Hansen E.B. (2018). Dissection of the antimicrobial and hemolytic activity of Cap18: Generation of Cap18 derivatives with enhanced specificity. PLoS ONE.

[109] Guo X., Yan T., Rao J., Yue X., Pei X., Deng J., Sun W., Yang W., Zhang B., Xie J. (2021). Potent Antimicrobial and Antibiofilm Activities of Feleucin-K3 Analogs Modified by α-(4-Pentenyl)-Ala against Multidrug-Resistant Bacteria. Biomolecules.

[110] Oddo A., Hansen P.R. (2017). Hemolytic Activity of Antimicrobial Peptides. Methods Mol. Biol..

[111] Souza J.G.S., Bertolini M., Costa R.C., Cordeiro J.M., Nagay B.E., de Almeida A.B., Retamal-Valdes B., Nociti F.H., Feres M., Rangel E.C. (2020). Targeting Pathogenic Biofilms: Newly Developed Superhydrophobic Coating Favors a Host-Compatible Microbial Profile on the Titanium Surface. ACS App. Mater. Interfaces.

[112] Piller P., Wolinski H., Cordfunke R.A., Drijfhout J.W., Keller S., Lohner K., Malanovic N. (2022). Membrane Activity of LL-37 Derived Antimicrobial Peptides against *Enterococcus hirae*: Superiority of SAAP-148 over OP-145. Biomolecules.

[113] Greco I., Molchanova N., Holmedal E., Jenssen H., Hummel B.D., Watts J.L., Håkansson J., Hansen P.R., Svenson J. (2020). Correlation between hemolytic activity, cytotoxicity and systemic in vivo toxicity of synthetic antimicrobial peptides. Sci. Rep..

[114] Gopal R., Kim Y.G., Lee J.H., Lee S.K., Chae J.D., Son B.K., Seo C.H., Park Y. (2014). Synergistic effects and antibiofilm properties of chimeric peptides against multidrug-resistant Acinetobacter baumannii strains. Antimicrob. Agents Chemother..

[115] de la Fuente-Nuñez C., Reffuveille F., Mansour S.C., Reckseidler-Zenteno S.L., Hernandez D., Brackman G., Coenye T., Hancock R.E.W. (2015). D-enantiomeric peptides that eradicate wild-type and multidrug-resistant biofilms and protect against lethal *Pseudomonas aeruginosa* infections. Chem. Biol..

[116] Jorge P., Grzywacz D., Kamysz W., Lourenço A., Pereira M.O. (2017). Searching for new strategies against biofilm infections: Colistin-AMP combinations against *Pseudomonas aeruginosa* and *Staphylococcus aureus* single- and double-species biofilms. PLoS ONE.

[117] Swedan S., Shubair Z., Almaaytah A. (2019). Synergism of cationic antimicrobial peptide WLBU2 with antibacterial agents against biofilms of multi-drug resistant *Acinetobacter baumannii* and *Klebsiella pneumoniae*. Infect. Drug Resist..

[118] Kalsy M., Tonk M., Hardt M., Dobrindt U., Zdybicka-Barabas A., Cytrynska M., Vilcinskas A., Mukherjee K. (2020). The insect antimicrobial peptide cecropin A disrupts uropathogenic *Escherichia coli* biofilms. NPJ Biofilms Microbiomes.

[119] Duong L., Gross S.P., Siryaporn A. (2021). Developing antimicrobial synergy with AMPs. Front. Med. Technol..

[120] Van Praagh A.D.G., Li T., Zhang S., Arya A., Chen L., Zhang X.X., Bertolami S., Mortin L.I. (2011). Dapptomucin antibiotic lock therapy in a rat model of staphylococcal central venous catheter biofilm interactions. Antimicrob. Agents Chemother..

[121] Signorino C., Fusco E., Galli L., Chiappini E. (2023). Effectiveness of Antimicrobial Lock Therapy for the Treatment of Catheter-Related and Central-Line-Associated Bloodstream Infections in Children: A Single Center Retrospective Study. Antibiotics.

[122] Riool M., de Beij A., Drijfhout J.W., Nibbering P.H., Zaat S.A.J. (2017). Antimicrobial peptides in biomedical device manufacturing. Front. Chem..

[123] Negut I., Bita B., Groza A. (2022). Polymeric Coatings and Antimicrobial Peptides as Efficient Systems for Treating Implantable Medical Devices Associated-Infections. Polymers.

[124] Copling A., Akantibila M., Kumaresan R., Fleischer G., Cortes D., Tripathi R.S., Carabetta V.J., Vega S.L. (2023). Recent Advances in Antimicrobial Peptide Hydrogels. Int. J. Mol. Sci..

[125] Andersen M.J., Flores-Mireles A.L. (2020). Urinary Catheter Coating Modifications: The Race against Catheter-Associated Infections. Coatings.

[126] Gefter Shenderovich J., Zaks B., Kirmayer D., Lavy E., Steinberg D., Friedman M. (2018). Chlorhexidine sustained-release varnishes for catheter coating—Dissolution kinetics and antibiofilm properties. Eur. J. Pharm. Sci..

[127] Menezes F.G., Correa L., Medina-Pestana J.O., Aguiar W.F., Camargo L.F.A. (2019). A randomized clinical trial comparing Nitrofurazone-coated and uncoated urinary catheters in kidney transplant recipients: Results from a pilot study. Transpl. Infect. Dis..

[128] Srisang S., Nasongkla N. (2019). Spray coating of foley urinary catheter by chlorhexidine-loadedpoly(epsilon-caprolactone) nanospheres: Effect of lyoprotectants, characteristics, and antibacterial activity evaluation. Pharm. Dev. Technol..

[129] Yu K., Lo J.C., Yan M., Yang X., Brooks D.E., Hancock R.E., Lange D., Kizhakkedathu J.N. (2017). Anti-adhesive antimicrobial peptide coating prevents catheter associated infection in a mouse urinary infection model. Biomaterials.

[130] Monteiro C., Costa F., Pirttila A.M., Tejesvi M.V., Martins M.C.L. (2019). Prevention of urinary catheter-associated infections by coating antimicrobial peptides from crowberry endophytes. Sci. Rep..

[131] Subramanian S., Huiszoon R.C., Chu S., Bentley W.E., Ghodssi R. (2020). Microsystems for biofilm characterization and sensing—A review. Biofilm.

[132] Brun-Buisson C., Abrouk F., Legrand P., Huet Y., Larabi S., Rapin M. (1987). Diagnosis of central venous catheter-related sepsis. Critical level of quantitative tip cultures. Arch. Intern. Med..

[133] Clinical and Laboratory Standards Institute (CLSI) (2017). Performance Standards for Antimicrobial Susceptibility Testing.

[134] The European Committee on Antimicrobial Susceptibility Testing (EUCAST) (2017). European Society of Clinical Microbiology and Infectious Diseases.V.2.0..

[135] O’Toole G.A. (2011). Microtiter dish biofilm formation assay. J. Vis. Exp..

[136] Hiemstra H.S., Duinkerken G., Benckhuijsen W.E., Amons R., de Vries R.R.P., Roep B.O., Drijfhout J.W. (1997). The identification of CD4^+^ T cell epitopes with dedicated synthetic peptide libraries. Proc. Natl. Acad. Sci. USA.

[137] Nell M.J., Tjabringa G.S., Wafelman A.R., Verrijk R., Hiemstra P.S., Drijfhout J.W., Grote J.J. (2006). Development of novel LL-37 derived antimicrobial peptides with LPS and LTA neutralizing and antimicrobial activities for therapeutic application. Peptides.

[138] Paduszynska M.A., Greber K.E., Paduszynski W., Sawicki W., Kamysz W. (2020). Activity of temporin A and short lipopeptides combined with gentamicin against biofilm formed by *Staphylococcus aureus* and *Pseudomonas aeruginosa*. Antibiotics.

[139] Compain F., Babosan A., Brisse S., Genel N., Audo J., Ailloud F., Kassis-Chikhani N., Arlet G., Decré D. (2014). Multiplex PCR for detection of seven virulence factors and K1/K2 capsular serotypes of *Klebsiella pneumoniae*. J. Clin. Microbiol..

[140] Shah R.K., Ni Z.H., Sun X.Y., Wang G.Q., Li F. (2017). The Determination and Correlation of Various Virulence Genes, ESBL, Serum Bactericidal Effect and Biofilm Formation of Clinical Isolated Classical *Klebsiella pneumoniae* and Hypervirulent *Klebsiella pneumoniae* from Respiratory Tract Infected Patients. Pol. J. Microbiol..

[141] El Fertas-Aissani R., Messai Y., Alouache S., Bakour R. (2013). Virulence profiles and antibiotic susceptibility patterns of *Klebsiella pneumoniae* strains isolated from different clinical specimens. Pathol. Biol..

[142] Cruz-Córdova A., Esteban-Kenel V., Espinosa-Mazariego K., Ochoa S.A., Espinosa S.M., de la Garza Elhain A., Rendón E.F., Villegas E.O.L., Xicohtencatl-Cortes J. (2014). Pathogenic determinants of clinical *Klebsiella pneumoniae* strains associated with their persistence in the hospital environment. Bol. Med. Hosp. Infant. Mex..

[143] Yun K.W., Kim H.Y., Park H.K., Kim W., Lim I.S. (2014). Virulence factors of uropathogenic *Escherichia coli* of urinary tract infections and asymptomatic bacteriuria in children. J. Microbiol. Immunol. Infect..

[144] Pal M., Singh S. (2007). PCR based detection of adhesive curli gene “crl” and ‘csgA’ in avian pathogenic Escherichia coli. Indian J. Anim. Res..

[145] Moulin-Schouleur M., Répérant M., Laurent S., Brée A., Mignon-Grasteau S., Germon P., Rasschaert D., Schouler C. (2007). Extraintestinal pathogenic *Escherichia coli* strains of avian and human origin: Link between phylogenetic relationships and common virulence patterns. J. Clin. Microbiol..

[146] Liu H., Wu Y.Q., Chen L.P., Gao X., Huang H.N., Qiu F.L., Wu D.C. (2016). Biofilm-Related Genes: Analyses in Multi-Antibiotic Resistant *Acinetobacter Baumannii* Isolates from Mainland China. Med. Sci. Monit..

[147] Fallah A., Rezaee M.A., Hasani A., Barhaghi M.H.S., Kafil H.S. (2017). Frequency of *bap* and *cpaA* virulence genes in drug resistant clinical isolates of *Acinetobacter baumannii* and their role in biofilm formation. Iran. J. Basic. Med. Sci..

