# Supplementary material for: Novel Antibacterial Agents SAAP-148 and Halicin Combat Gram-Negative Bacteria Colonizing Catheters
Source: Antibiotics (Basel). 2023 Dec 16;12(12):1743. doi: 10.3390/antibiotics12121743 (PMC10741160; doi:10.3390/antibiotics12121743)
Supplement: Supplementary file 1 [file antibiotics-12-01743-s001.zip › antibiotics-2741122-supplementary.pdf]

Supplementary materials

Supplementary table S1. Antibiotics tested in this study

Strains	Antibiotic	Concentration (μg/mL)
Enterobacteriaceae	Amoxicillin-clavulanic acid	20+10
	Ceftazidime	30
	Cefotaxime	30
	Cefepim	30
	Cefoxitin	30
	Gentamycin	10
	Imipenem	10
	Ciprofloxacin	5
	Tetracycline	30
	Nalidixic acid	30
	Co-trimoxazole	25
***Acinetobacter* spp.**	Ceftazidime	30
	Cefotaxime	30
	Cefepim	30
	Cefoxitin	30
	Gentamycin	10
	Imipenem	10
	Ciprofloxacin	5
	Tetracycline	30
	Co-trimoxazole	25

Supplementary table S2. Target genes and primers used in this study.

Target organism	Gene name	Gene function	Forward primer	Reverse primer	Product (bp)	Reference
Klebsiella pneumoniae	*mrkD*	Adhesin type 3 fimbriae	AAGCTATCGCTGTACTTCCGGCA	GGCGTTGGCGCTCAGATAGG	340	[139]
	fimH-1	Adhesin type1 fimbriae	ATGAACGCCTGGTCCTTTGC	GCTGAACGCCTATCCCCTGC	688	[140]
	ycfM	Outer membrane lipoprotein	ATCAGCAGTCGGGTCAGC	CTTCTCCAGCATTCAGCG	160	[141]
	ecpA	*E. coli*	AATGGTTCACCGGGACATCA	AAGGATGAAATATCGCCGAC	759	[142]

		common pilus	TGTC	ATCC		
E. coli	*fimH*	Fimbiral adhesins	TGCAGAACGGATAAGCCGTG	GCAGTCACCTGCCCTCCGGTA	508	[143]
	csgA	Curli adhesin	ACTCTGACTTGACTATTACC	AGATGCAGTCTGGTCAAC	200	[144]
	hlyF	Putative hemolysin	TCGTTTAGGGTGCTTACCTTCAAC	TTTGGCGGTTTAGGCATTCC	444	[145]
A. baumannii	*csuE*	Adhesins	ATGCATGTTCTCTGGACTGATGTTGAC	CGACTTGTACCGTGACCGTATCTTGATAAG	976	[146]
	ompA	efflux system	CAATTGTTATCTCTGGAG	ACCTTGAGTAGACAAACGA	966	
	bap	biofilm-associated protein	TAG GGA GGG TAC CAA TGC AG	TCA TGA TTT GAT GCT GCA GCG ATA A	400	[147]

Supplementary table S3. PCR programs for virulence genes detection

Gene	Initial denaturation temperature (time)	Denaturation temperature (time)	Annealing temperature (time)	Extension temperature (time)	Number of cycles	Final extension temperature (time)	Final concentration (µM)
mrkD	95°C (5min)	94°C (30s)	60°C (90s)	72°C (1min)	30	72°C (10min)	0.1
fimH-1<br>***ycfM***<br>***ecpA***	95°C (4min)	95°C (45s)	55°C (1min)	72°C (1min)	30	72°C (5min)	0.4
fimH	94°C (5 min)	94°C (60s)	55°C (60s)	72°C (2min)	35	72°C (10min)	
csgA	95°C (3 min)	94°C (1min)	48°C (1min)	72°C (1min)	30	72°C (5min)	0.4
hlyF	94°C (3 min)	94°C (1 min)	51°C (60s)	72°C (1 min)	30	72°C (10 min)	
csuE	95°C for 10min	95°C for 30s	63°C for 30s	72°C for 1min	35	72°C for 10min	
ompA	95°C for 10min	95°C for 30s	52°C for 30s	72°C for 1min	35	72°C for 10min	

bap	95°C for 2min	95°C for 30s	61°C for 30s	68°C for 1min	30	68°C for 2min

*Modifications have been made in the PCR program

Supplementary table S4. Concentrations of SAAP-148 and halicin tested in the checkerboard

**Strain**	**24-hrs treatment**			
	**Final concentration SAAP-148 (µM)**	**Final concentration Halicin (µM)**	**Control SAAP-148(µM)**	**Control Halicin (µM)**
***E. coli* EC2**	3.2-25.6	3.2-12.8	3.2-102.4	3.2-51.2
***A. baumannii* AB1**	3.2-25.6	12.8-51.2	3.2-102.4	12.8-204.8
***K. pneumoniae* KP1**	3.2-25.6	12.8-102.4	3.2-102.4	12.8-204.8
***K. pneumoniae* KP2**	3.2-25.6	12.8-102.4	3.2-102.4	12.8-204.8

References

139 Compain, F.; Babosan, A.; Brisse, S.; Genel, N.; Audo, J.; Ailloud, F.; Kassis-Chikhani, N.; Arlet, G.; Decré, D. Multiplex PCR for detection of seven virulence factors and K1/K2 capsular serotypes of *Klebsiella pneumoniae*. *J. clin. Microbiol*. **2014**, 52, 4377–4380. https://doi.org/10.1128/JCM.02316-14

140 Shah, R. K.; Ni, Z. H.; Sun, X. Y.; Wang, G. Q.; Li, F. The Determination and Correlation of Various Virulence Genes, ESBL, Serum Bactericidal Effect and Biofilm Formation of Clinical Isolated Classical *Klebsiella pneumoniae* and Hypervirulent *Klebsiella pneumoniae* from Respiratory Tract Infected Patients. *Pol. J. Microbiol*, **2017**, 66, 501–508. https://doi.org/10.5604/01.3001.0010.7042

141 El Fertas-Aissani, R.; Messai, Y.; Alouache, S.; Bakour, R. Virulence profiles and antibiotic susceptibility patterns of *Klebsiella pneumoniae* strains isolated from different clinical specimens. *Pathol. Biol (Paris)*. **2013**, 61, 209–216. https://doi.org/10.1016/j.patbio.2012.10.004

142 Cruz-Córdova, A.; Esteban-Kenel, V.; Espinosa-Mazariego, K.; Ochoa, S.A.; Espinosa, S.M.; de la Garza Elhain, A.; Rendón, E.F.; Villegas, E.O.L.; Xicohtencatl-Cortes, J. Pathogenic determinants of clinical *Klebsiella pneumoniae* strains associated with their persistence in the hospital environment. *Bol. Med. Hosp. Infant. Mex*. **2014**, 71, 1.

143 Yun, K. W.; Kim, H. Y.; Park, H. K.; Kim, W., Lim, I. S. Virulence factors of uropathogenic *Escherichia coli* of urinary tract infections and asymptomatic bacteriuria in children. *J. Microbiol. Immunol. Infect*. **2014**, 47, 455–461. https://doi.org/10.1016/j.jmii.2013.07.010

144 Pal, M.; Singh, S. PCR based detection of adhesive curli gene "crl" and 'csgA' in avian pathogenic *Escherichia coli*. *Indian J. Anim. Res*. **2007**, 41, 226–229.

145 Moulin-Schouleur, M.; Répérant, M.; Laurent, S.; Brée, A.; Mignon-Grasteau, S.; Germon, P.; Rasschaert, D.; Schouler, C. Extraintestinal pathogenic *Escherichia coli* strains of avian and human origin: link between phylogenetic relationships and common virulence patterns. *J. Cli. Microbiol*. **2007**, 45, 3366–3376. https://doi.org/10.1128/JCM.00037-07

146 Liu, H.; Wu, Y. Q.; Chen, L. P.; Gao, X.; Huang, H. N.; Qiu, F. L.; Wu, D. C. Biofilm-Related Genes: Analyses in Multi-Antibiotic Resistant *Acinetobacter Baumannii* Isolates From Mainland China. *Med. Sci. Monit*. **2016**, 22, 1801–1807. https://doi.org/10.12659/msm.898959

147 Fallah, A.; Rezaee, M. A.; Hasani, A.; Barhaghi, M. H. S.; Kafil, H. S. Frequency of *bap* and *cpaA* virulence genes in drug resistant clinical isolates of *Acinetobacter baumannii* and their role in biofilm formation. *Iran. J. Basic. Med. Sci*. **2017**, 20, 849–855. https://doi.org/10.22038/IJBMS.2017.9105
